# The proteome of *Nicotiana benthamiana* is shaped by extensive protein processing

**DOI:** 10.1111/nph.19891

**Published:** 2024-06-09

**Authors:** Kaijie Zheng, Joy C. Lyu, Emma L. Thomas, Mariana Schuster, Nattapong Sanguankiattichai, Sabrina Ninck, Farnusch Kaschani, Markus Kaiser, Renier A. L. van der Hoorn

**Affiliations:** ^1^ Key Laboratory of Soybean Molecular Design Breeding, Northeast Institute of Geography and Agroecology Chinese Academy of Sciences Changchun 130102 China; ^2^ The Plant Chemetics Laboratory, Department of Biology University of Oxford Oxford OX1 3RD UK; ^3^ Chemical Biology, Center of Medical Biotechnology (ZMB), Faculty of Biology University of Duisburg‐Essen Essen 45141 Germany

**Keywords:** agroinfiltration, molecular weight, *Nicotiana benthamiana*, protein migration, protein processing

## Abstract

- Processing by proteases irreversibly regulates the fate of plant proteins and hampers the production of recombinant proteins in plants, yet only few processing events have been described in agroinfiltrated *Nicotiana benthamiana*, which has emerged as the main transient protein expression platform in plant science and molecular pharming.
- Here, we used in‐gel digests and mass spectrometry to monitor the migration and topography of 5040 plant proteins within a protein gel. By plotting the peptides over the gel slices, we generated peptographs that reveal where which part of each protein was detected within the protein gel.
- These data uncovered that 60% of the detected proteins have proteoforms that migrate at lower than predicted molecular weights, implicating extensive proteolytic processing. This analysis confirms the proteolytic removal and degradation of autoinhibitory prodomains of most but not all proteases, and revealed differential processing within pectinemethylesterase and lipase families. This analysis also uncovered intricate processing of glycosidases and uncovered that ectodomain shedding might be common for a diverse range of receptor‐like kinases. Transient expression of double‐tagged candidate proteins confirmed processing events *in vivo*.
- This large proteomic dataset implicates an elaborate proteolytic machinery shaping the proteome of *N. benthamiana*.

## Introduction

Agroinfiltration of *Nicotiana benthamiana* is routinely used in plant science and has emerged as a powerful protein expression platform in molecular pharming. Yet, the yields or recombinant protein production are hampered by processing and degradation caused by endogenous plant proteases. The *N. benthamiana* genome encodes for > 1200 putative proteases (Jutras *et al*., 2020), but little is known about their endogenous substrates. Most processing events may inactivate proteins, but protein cleavages can also regulate proteins for instance by releasing active proteins from their precursors (Dissmeyer *et al*., 2018). Protein processing can also change the subcellular location of proteins or their ability to interact with other components inside and outside the cell. Understanding protein processing in plants is limited because the techniques available for studying protein processing are not yet well‐developed.

Several global studies on protein processing include the use of shotgun proteomics on isotope‐labeled Arabidopsis cell cultures to monitor life times of plant proteins (Nelson *et al*., 2014); the use of Terminal Amine Isotope Labelling of Samples (TAILS), for example to monitor the role of PRT6 in the N‐terminome of *Arabidopsis thaliana* (Zhang *et al*., 2018); the use of High‐efficiency Undecanal‐based N Termini EnRichment (HUNTER), for example to identify putative substrates of vacuolar processing enzymes in Arabidopsis seeds (Weng *et al*., 2019); and the use of COmbined FRactional DIagonal Chromatography (COFRADIC), for example to identify putative substrates of metacaspase‐9, based on the detection of N‐termini (Tsiatsiani *et al*., 2013). These, and similar powerful proteomic methods, resulted in many candidate protease substrates that remain to be confirmed. Candidate substrates identified by TAILS, COFRADIC and shotgun proteomics are challenging to confirm because neo‐termini can originate from proteins that are only incompletely processed or already degraded. In addition, neo‐termini that appear near the termini of proteins are difficult to confirm by detecting shifts in molecular weight (MW).

Here, we take a different, complementary approach to identify candidate protease substrates by a shift in migration in protein gels. We use the PRotein TOpography and Migration Analysis Platform (PROTOMAP) to identify proteins that migrate in the protein gel at a different than predicted MW. PROTOMAP is a proteomic analysis approach that charts tryptic peptides detected by mass spectrometry (MS) from gel slices of a 1D‐SDS‐PAGE gel in two dimensions: the protein sequence (*x*‐axis) and the gel slice at which the peptide was detected (y‐scale) (Dix *et al*., 2008). By including MS data from all gel slices of an 1D‐gel and including replicates, this PROTOMAP approach results in a global overview of where within the protein gel, which part of the protein is migrating. This analysis generates the same type of information as Western blots, but then for all detectable proteins simultaneously. Thus, in contrast to other proteomic techniques, PROTOMAP will display changes in the migration of proteins within protein gels.

PROTOMAP was originally used to identify 261 processing events during apoptosis (Dix *et al*., 2008) and was used to demonstrate that phosphorylation sculpts the apoptotic proteome (Dix *et al*., 2012). PROTOMAP has also been used to profile protein processing during infection with the malaria parasite (Bowyer *et al*., 2011) but has not been used in plant science because of the high demand on machine time. For instance, the generation of PROTOMAP data for a single proteome involves the analysis of *c*. 100 in‐gel tryptic digests by MS.

Here, we sought to generate a PROTOMAP dataset from agroinfiltrated *N. benthamiana*, taking advantage of its recently improved genome annotation (Ranawaka *et al*., 2023). Transient expression by agroinfiltration of *N. benthamiana* is frequently used in plant science to study, for example subcellular localization and protein–protein interactions, and in molecular pharming to produce pharmaceutical proteins and metabolites (Eidenberger *et al*., 2023). However, the proteolytic machinery of *N. benthamiana* includes > 1000 putative proteases that hamper molecular pharming and research in plant science (Jutras *et al*., 2020), and therefore, the characterization of protein processing in agroinfiltrated leaves will benefit both plant science and molecular pharming. In addition, by analyzing processing in agroinfiltrated leaves, we should be able to verify processing events upon transient expression of reporter‐tagged precursor proteins.

## Materials and Methods

### Plant growth and agroinfiltration

*Nicotiana benthamiana* Domin (LAB strain) plants were grown in a growth cabinet under long‐day cycle with 16 h : 8 h, 22°C : 20°C, light : dark (80 μmol m^−2^ s^−1^). This experiment was originally designed to be the control to detect the earliest protein processing events during the hypersensitive response (HR) triggered by co‐expressing potato *Rx* with the coat protein of PVX, which induces tissue collapse at 21 h postagroinfiltration (21 hpa), but the chosen time point (17 hpa) was too early to detect HR‐related processing or even to detect Rx or CP by LC‐MS/MS (Supporting Information Notes S1). The control samples used for this experiment consisted of a 1 : 1 mixture of frozen leaf powders from leaves expressing *Rx* with leaves expressing *CP*.

*Agrobacterium tumefaciens* Conn C58C1 containing binary vectors with either *Rx* from *Solanum tuberosum* L. or Coat Protein from Potato Virus X (Bendahmane *et al*., 1999) were grown overnight in Luria–Bertani medium supplemented with 100 μg ml^−1^ rifampicin and kanamycin at 28°C with 220 rpm overnight. Cultures were pelleted and washed with infiltration buffer (10 mM MES, 10 mM MgCl_2_, pH 5.7). Cells were resuspended in infiltration buffer to final OD_600_ 0.5 and acetosyringone added to 100 μM. The top two fully expanded leaves of 4–5‐wk‐old plants were agroinfiltrated with either Rx or CP alone (non‐HR samples). Leaves were harvested at 17 h postinfiltration and ground with liquid nitrogen. Ground frozen tissue from non‐HR‐induced leaves agroinfiltrated with *Rx* or *CP* alone was mixed in a 1 : 1 ratio.

### Protein extraction and gel electrophoresis

Extraction buffer (100 mM HEPES pH 7.5, 5 mM DTT, MS SAFE Protease and Phosphatase Inhibitor (Sigma)) and leaf powder were mixed in a 1 : 1 ratio (i.e. 300 μg powder in 300 μl buffer). Extract was sonicated (Diagenode Biorupter) at full power for 10 min at 4°C. The samples were cleared by centrifugation at 11 000 ***g*** for 20 min at 4°C and the retained supernatant centrifuged for a further 5 min. Protein concentration was determined using Bradford Assay, and the cleared lysate was mixed with lithium dodecyl sulfate loading buffer (Thermo Fisher Scientific, Waltham, MA, USA) and DTT to a final concentration of 200 mM and then boiled at 90°C for 10 min. Fifty micrograms of sample was then loaded onto an SDS‐PAGE pre‐cast 4–12% Bis–Tris gel (Thermo Fisher Scientific) and separated with MOPS buffer (Thermo Fisher Scientific). Each sample lane was sliced into 24 2.5‐mm sections guided by a background grid (Fig. S1). The migration of protein ladder (Multicolor Broad Range Protein Ladder; Thermo Scientific) was used to estimate MWs of proteins in each gel slice (Fig. S1).

Gel slices were washed twice in HPLC grade water and then in 100 mM ammonium bicarbonate. Proteins were reduced by incubation with 10 mM TCEP at 62°C for 30 min and then alkylated in 55 mM iodoacetamide for 30 min in the dark. Gel slices were dehydrated by four washes in 50% acetonitrile (ACN) and a final wash in 100% ACN. Remaining solution was removed in a vacuum centrifuge (Eppendorf, Hamburg, Germany). Proteins were then digested with the addition of 400 ng of trypsin in 25 mM ammonium bicarbonate and incubation overnight at 37°C. All solutions were prepared in 100 mM ammonium bicarbonate. Peptide containing solution was eluted by successive addition of 5% formic acid in ACN washes. Peptides were dried in a vacuum centrifuge (Eppendorf) and desalted on home‐made C18 stage tips as previously described (Rappsilber *et al*., 2007). Peptides were resuspended in 0.1% (v/v) formic acid for loading onto the LC‐MS/MS.

### Proteomic analysis

Samples were analyzed with an Orbitrap Elite (Thermo Fischer Scientific) coupled to an Easy‐nLC 1000 liquid chromatography (LC) system (Thermo Fisher Scientific) (Michalski *et al*., 2012). The LC was run in a single column mode with an analytical column of a fused silica capillary (75 μm × 22 cm) with an integrated PicoFrit emitter (New Objective, Littleton, MA, USA) packed in‐house with Reprosil‐Pur 120 C18‐AQ 1.9 μm resin (Dr. Maisch). The analytical column was encased by a column oven (PRSO‐V1; Sonation) at 45°C and attached to a nanospray flex ion source (Thermo Fisher Scientific). All solvents were of UPLC grade (Sigma‐Aldrich). Peptides were directly loaded onto the analytical column with a maximum flow rate that would not exceed the set pressure limit of 980 bar (usually *c*. 0.6–1.0 μl min^−1^). Peptides were separated by running a 70‐min gradient of solvent A and solvent B (start with 7% B; gradient 7–35% B for 60 min; gradient 35–80% B for 5 min and 80% B for 5 min) at a flow rate of 300 nl min^−1^.

The Xcalibur software (v.2.2 SP1.48) was used for data acquisition with the mass spectrometer in positive ion mode. Precursor ion scanning was performed in the Orbitrap analyzer (FTMS; Fourier Transform Mass Spectrometry) in the scan range of *m/z* 300–1500 and at a resolution of 60 000 with the internal lock mass option turned on (lock mass was 445.120025 *m*/*z*, polysiloxane) (Olsen *et al*., 2005). Product ion spectra were recorded in a data‐dependent fashion in the ion trap (ITMS) in a variable scan range and at a rapid scan rate (wideband activation was turned on). The ionization potential (spray voltage) was set to 1.8 kV. Peptides were analyzed using a repeating cycle consisting of a full precursor ion scan (3.0 × 10^6^ ions or 50 ms) followed by 12 product ion scans (1.0 × 10^4^ ions or 50 ms) where peptides are isolated based on their intensity in the full survey scan (threshold of 500 counts) for tandem mass spectrum (MS2) generation that permits peptide sequencing and identification. Collision‐induced dissociation energy was set to 35% for the generation of MS2 spectra. During MS2 data acquisition, dynamic ion exclusion was set to 120 s with a maximum list of excluded ions consisting of 500 members and a repeat count of one. Ion injection time prediction, preview mode for the FTMS, monoisotopic precursor selection and charge state screening were enabled. Only charge states higher than 1 were considered for fragmentation.

Raw spectra were analyzed with Andromeda search engine (Cox *et al*., 2011) in Max Quant v.1.5.5.30 (Cox & Mann, 2008). Primarily default parameters were selected including LFQ and match between runs. Spectra were searched against the LAB3.60 *N. benthamiana* proteome database (Ranawaka *et al*., 2023). Permitted modifications included static carbamidomethylation of cysteines, variable N‐terminal acetylation and methionine oxidation and trypsin specific digestion. The instrument type in Andromeda searches was set to Orbitrap, and the precursor mass tolerance was set to ±20 ppm (first search) and ±4.5 ppm (main search). The MS/MS match tolerance was set to ±0.5 Da. The peptide spectrum match FDR and the protein FDR were set to 0.01 (based on target‐decoy approach). Minimum peptide length was seven amino acids. For protein quantification, unique and razor peptides were allowed. Modified peptides were allowed for quantification. The minimum score for modified peptides was 40. Label‐free protein quantification was switched on, and unique and razor peptides were considered for quantification with a minimum ratio count of 2. Retention times were recalibrated based on the built‐in nonlinear time‐rescaling algorithm. MS/MS identifications were transferred between LC‐MS/MS runs with the ‘match between runs’ option in which the maximal match time window was set to 0.7 min and the alignment time window set to 20 min. The quantification is based on the ‘value at maximum’ of the extracted ion current. At least two quantitation events were required for a quantifiable protein.

### PROTOMAP analysis

Peptide reads were analyzed in R with customized scripts (Notes S3). Contaminants and non‐plant sequences were removed before any processing. To suppress noise signals of ions that were detected across many gel slices caused by high ionization efficiencies in contrast to other peptides of the same proteins, we removed peptides from gel slices that were detected at < 10% of the total ion intensity of that peptide across all gel slices. For each protein, peptides were plotted along the sequence across each gel slice on peptograph with ggplot2 package (Wickham, 2011). The number of peptides from each gel slice was plotted alongside as a reference of signal strength from the slice. Signal peptides, transmembrane domains and Pfam domain annotation were predicted by SignalP5.0 (Teufel *et al*., 2022), Tmhmm (Krogh *et al*., 2001) and InterPro (Mistry *et al*., 2021), respectively, and mapped along the sequences. MWs of proteins were predicted using the Peptides package (Osorio *et al*., 2015). The sequons were predicted by scanning through the sequence for NxS/T, and an additional 3 kDa was added for each sequon to the predicted MW to calculate the *N*‐glycosylated MW.

### Molecular cloning

To generate the double‐tagged binary cloning vector, the *2x35S* promoter and *SP*(*PR1a*)‐intron fusion, a fragment encoding FLAG‐RFP and a fragment encoding 2xHis‐GFP fused to the *35S* terminator was amplified from template plasmids listed in Table S1 using primers listed in Table S2. The PCR products were digested with restriction enzymes listed in Table S2 and cloned into *pJK001c* (Paulus *et al*., 2020) using EcoRI and PmeI restriction enzymes, resulting in *pKZ45*, a binary vector carrying an open reading frame (ORF) encoding SP‐FLAG‐RFP‐2xHis‐GFP (SP‐FR‐HG). Full‐length ORFs of each target gene without the region encoding the endogenous signal peptide were amplified by PCR from cDNA generated from mRNA isolated from wild‐type (WT) *N. benthamiana* seedlings, using primers listed in Table S2. These fragments were cloned into *pKZ45* using restriction enzymes indicated in the primer names (Table S2), resulting in binary vectors for the expression of double‐tagged SCPL25 (pKZ47); EDA2 (pKZ49); GMC (pKZ54); PNGaseA (pKZ60); and SCPL20b (pKZ67), listed in Table S1.

### Western blot analysis

Total protein was isolated from *N. benthamiana* leaves at 3–5 d after agroinfiltration. Three 8‐mm‐diameter disks were taken per leaf and ground in liquid nitrogen. The frozen leaf powder was mixed with 200 μl 2 × gel loading buffer containing 100 mM Tris–HCl pH 6.8, 200 mM DTT, 4% SDS, 0.2% bromophenol blue and 20% glycerol. Samples were vortexed vigorously and immediately heated at 95°C for 8 min. Heated samples were briefly vortexed and centrifugated at 10 000 ***g*** for 1 min. Five microliters of supernatant was loaded onto 10% SDS polyacrylamide gels, and the proteins were transferred onto PVDF membranes using the *Trans*‐Blot Turbo RTA Transfer Kit (1704275, Bio‐rad). Membranes were blocked in PBS‐T (tablets 524650‐1EA; Sigma‐Aldrich) containing 0.05% Tween‐20 and 5% skim milk for 1 h, and then with antibody (1 : 5000 anti‐His‐HRP (130‐092‐785; Miltenyi Biotec, Bergish Gladbach, Germany) or anti‐FLAG‐HRP (Ab49763; Abcam Limited, Cambridge, UK)) overnight at 4°C and washed with PBS‐T three times and PBS one time. To detect the signals, the membrane was incubated with SuperSignal West Femto Maximum Sensitivity Substrate (34096; Thermo Fisher Scientific), and chemiluminescent signals were detected with ImageQuant LAS 4000 (GE Healthcare, Chalfont St Giles, UK).

### Phylogenetic analysis

Proteome sequences of *N. benthamiana* and *A. thaliana* were obtained from LAB3.60 (Ranawaka *et al*., 2023) and Araport11 (Cheng *et al*., 2017) annotations, respectively. Protein sequences from these proteomes were annotated for Pfam (Mistry *et al*., 2021) protein families and domains using InterProScan 5 (Jones *et al*., 2014). To select proteins to construct a phylogenetic tree for each family of interest, all proteins annotated with a PFAM ID of each family – serine carboxypeptidase‐like (SCPL) (PF00450), pectin methylesterase (PME) (PF01095) and GDSL‐like lipase/acylhydrolase (GELP) (PF00657) – were selected. Next, to identify additional family members that might have been missed, this Pfam‐identified protein set was used as a query to search against proteomes of both LAB3.60 and Araport11 using Blast+ (Camacho *et al*., 2009), keeping hits with higher than 80% query coverage. The Pfam‐identified and Blast‐identified proteins were combined to generate the final protein set for each family. Then, protein sequences were aligned using Mafft 7 (Katoh & Standley, 2013) with the L‐INS‐i algorithm. The resulting multiple sequence alignment was used to construct a maximum likelihood phylogenetic tree using Iq‐Tree 2 (Minh *et al*., 2020) with WAG amino acid substitution model, empirical amino acid frequencies (+F), allowance for invariant sites (+I) and Gamma‐distributed evolutionary rate heterogeneity among sites with four discrete categories (+G4). Branch support values were calculated using ultrafast bootstrap with 1000 replications. Phylogenetic trees were visualized using iTOL (Letunic & Bork, 2021). Trees were rooted at midpoint.

## Results

**Fig. 1:**
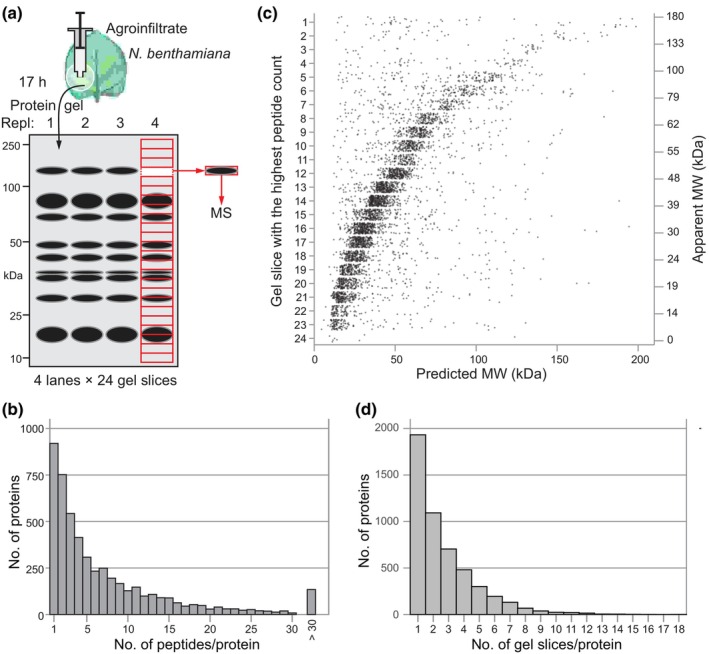
Detection of 5040 plant proteins in agroinfiltrated *Nicotiana benthamiana* leaves. (a) Total extracts from agroinfiltrated *N. benthamiana* leaves were separated by SDS‐PAGE and the lane was cut into 24 slices. Proteins in each gel slice were digested with trypsin and analyzed by LC‐MS/MS. The spectra detected in the replicates were combined for each gel slice for further analysis. (b) Number of peptides per protein. Spectra were mapped to the LAB3.60 annotation of the *N. benthamiana* proteome and the number of unique and non‐unique peptides were plotted for each protein. (c) Correlation between predicted and apparent molecular weight (MW). For each protein, the apparent MW was plotted against the predicted MW. The predicted MW was calculated after subtracting the putative signal peptide. (d) Number of gel slices in which each protein was detected, not including adjacent gel slices.

To generate the PROTOMAP dataset, we separated the soluble proteomes of extracts from agroinfiltrated leaves in adjacent lanes on a 4–12% gradient Bis–Tris protein gel and cut each lane into 24 gel slices, covering a range of 10–180 kDa indicated by the flanking marker lanes (Fig. S1). We digested the proteins in these gel slices with trypsin and analyzed the released peptides by LC‐MS/MS (Fig. 1a). We annotated the spectra to predicted tryptic peptides generated *in silico* from the recently released LAB3.60 annotation of the *N. benthamiana* genome (Ranawaka *et al*., 2023). The annotation to the LAB3.60 drastically increased the frequency of unique peptides when compared to our earlier NbDE database (Kourelis *et al*., 2019; Fig. S2). A total of 36 967 different tryptic peptides derived from 5040 plant proteins were detected in these 96 gel slices, making this the largest proteomics dataset available for agroinfiltrated *N. benthamiana* leaves (Notes S1). Almost 82% of the detected proteins were identified with more than one single peptide (Fig. 1b). The 919 proteins that were identified by a single peptide are retained in this analysis because their detection within the gel is often consistent with the predicted MW and the migration of homologous proteins (Fig. S3). All four replicates were combined per gel slice for further analysis.

We next plotted the predicted MW on the *x*‐axis against the gel slice with the highest number of peptides (*y*‐axis) for each protein. Since we are interested in secreted proteins, we also calculated the predicted MW of the protein without its SignalP‐predicted signal peptide (SP, Teufel *et al*., 2022). This graph shows a linear correlation between the predicted and apparent MW (*r* = 0.399, *P* = 2.2e^−16^) (Fig. 1c). Interestingly, there are also 1052 proteins from which most peptides were found at MW 10% below the predicted MW. Likewise, 1059 proteins have the highest peptide count at a MW 10% above the predicted MW. Another 59 proteins have their highest peptide count in Slice 1 (top slice), which is either caused by protein insolubility or the protein being too large to migrate into the polyacrylamide gel.

Most proteins (74.3%) are not detected in a single gel slice. For 79.5% of these 3744 proteins (2977 proteins), peptides from the same protein were detected in adjacent gel slices, which is presumably caused by diffuse protein signals within the protein gel. For 3011 proteins, however, peptides were also detected at two or more gel slices non‐adjacent from the gel slice with the main count (Fig. 1d), indicating that we are detecting multiple proteoforms for 59.7% of the 5040 detected proteins.

### Building an upgraded PROTOMAP dataset

**Fig. 2:**
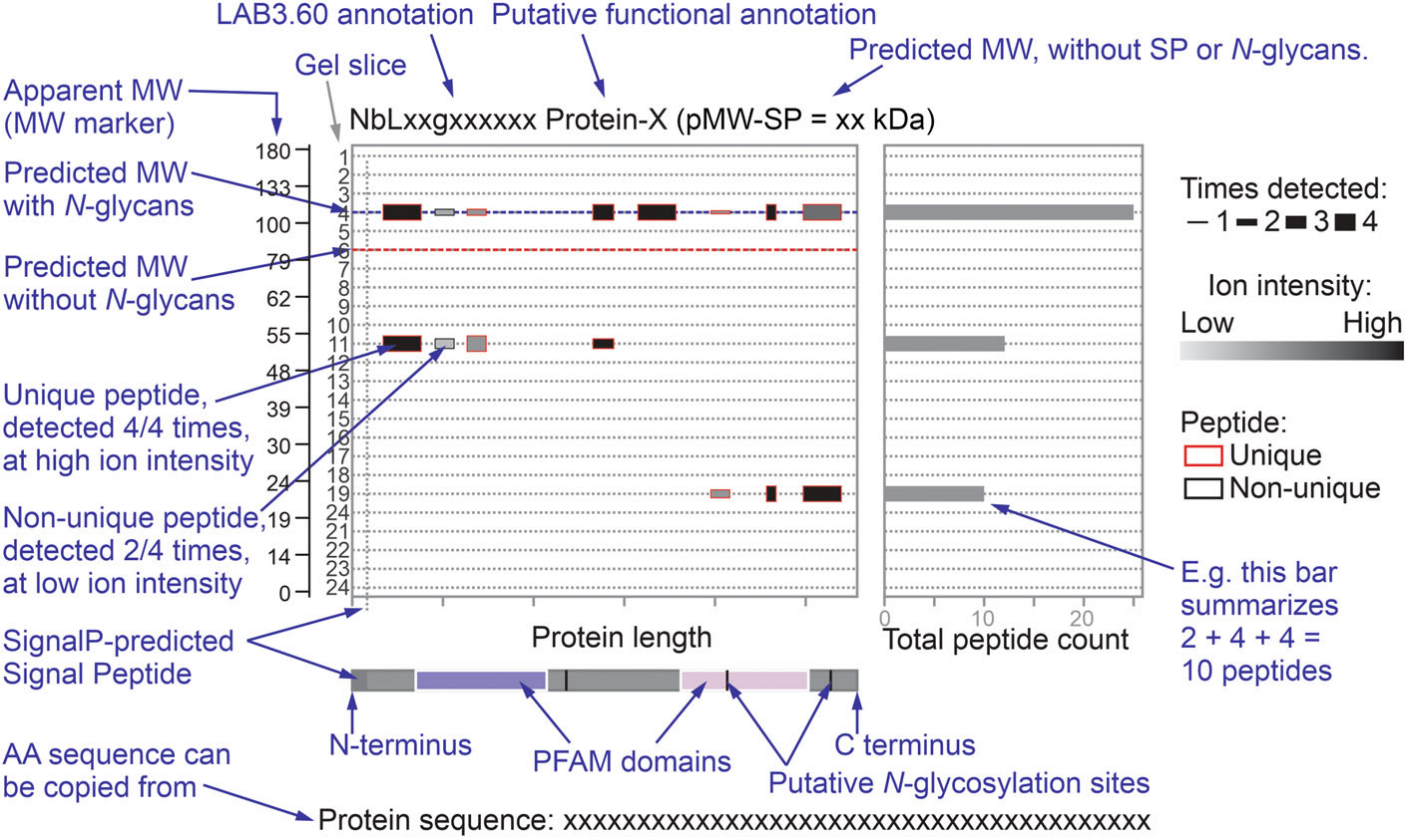
Peptographs display topology and migration for each protein. For each protein, the detected peptides were mapped onto the protein (*x*‐axis) for each gel slide (*y*‐axis). The number of peptides detected in a gel slice is summarized in the bar graph on the left. The apparent molecular weight (MW) marker estimated from the protein gel is added to the left. The relative ion intensities of the peptides are indicated in gray scale and the number of times of the four replicates they were detected is indicated by the height of the peptide box. Unique peptides have a red outline. The red dash line indicates the predicted MW without SignalP‐predicted signal peptide or predicted *N*‐glycans; the blue dash line indicates the MW without SP but with *N*‐glycans, based on the presence of sequons (NxS/T). The header on the top summarizes the protein identifier, putative function and predicted MW without SP and without predicted *N*‐glycans. The bar on the bottom represents the protein from N‐ to C‐terminus, with the Pfam domains, putative SP and transmembrane domain, and the position of the putative *N*‐glycosylation sites (NxS/T). The protein sequence is given at the bottom of the peptograph to be copied for further analysis.

To map the distributions of the peptides over the detected protein (*x*‐axis) and the gel slice (*y*‐axis), we produced an R script that generates peptographs for each protein, similar to how PROTOMAP was built originally (Dix *et al*., 2008), but with several important upgrades (Fig. 2). First, we highlight all unique peptides with red edges. Second, we depict in how many of the four replicates the peptide was detected in the thickness of the rectangle indicating the detected peptide to signify robustly detected peptides. The peptides are also depicted translucent to show partially overlapping peptides. Third, we depict the average signal intensity of each peptide using the gray scale to highlight peptides detected at higher ion intensities. Fourth, we added horizontal lines to indicate the gel slice in which the protein is expected to accumulate, based on the predicted MW for the protein without SignalP‐predicted signal peptide and predicted *N*‐glycans (red horizontal line) or with predicted *N*‐glycans (blue horizontal line), based on the presence of sequons (NxS/T, +3 kDa each). *O*‐glycosylation was not considered as it is less common and less predictable when compared to *N*‐glycosylation. Fifth, instead of the average spectral counts (Dix *et al*., 2008), we plotted the total peptide count for all four replicates for each gel slice in the graph on the right to indicate gel slices containing the strongest protein signals. Finally, we highlight the position of putative *N*‐glycosylation sites (NxS/T sequons) in the protein, together with InterPro‐predicted Pfam domains (Mistry *et al*., 2021), SignalP‐predicted signal peptides (Teufel *et al*., 2022) and Tmhmm‐predicted transmembrane domains (Krogh *et al*., 2001). And as with the original PROTOMAP, we provide the annotation of the putative protein function (top), an explanation of the Pfam domains and the protein sequence (bottom). These peptographs have been generated for every detected protein in a PDF format that can be searched for accession numbers, Pfam domains, keywords and sequences (Notes S2). The R script to produce these peptographs is provided as Notes S3.

### Secreted proteases accumulate as mature enzymes lacking autoinhibitory domains

**Fig. 3:**
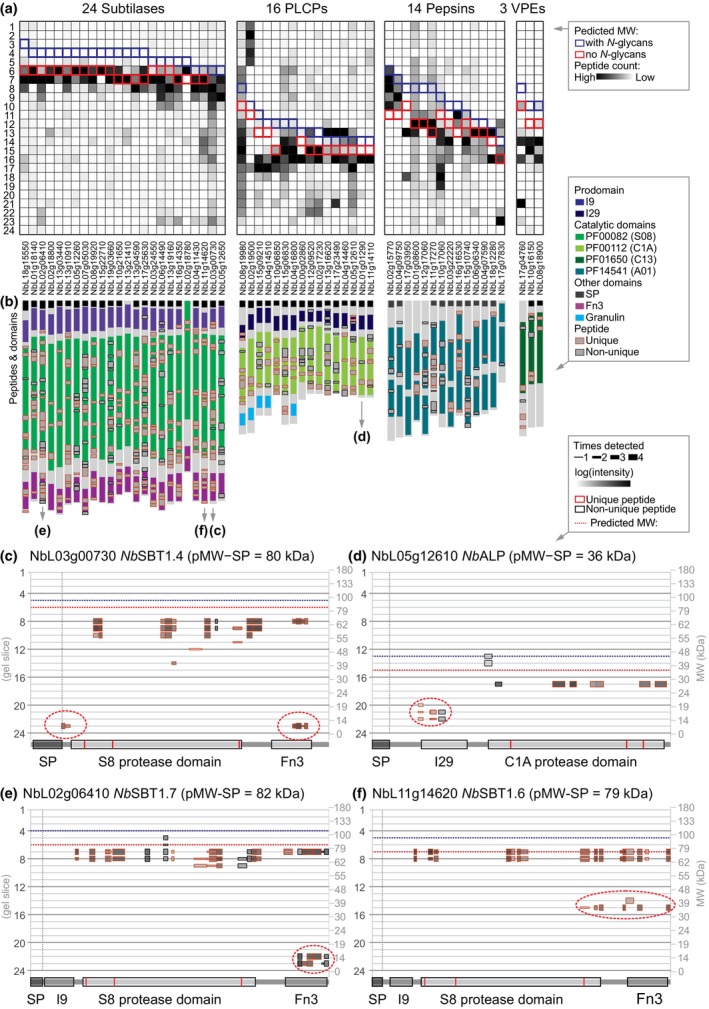
PROTOMAP displays migration and processing of mature proteases. (a) Peptographs of proteases with known autoinhibitory prodomains were extracted using Pfam families PF00082 (S08 proteases/subtilases); PF00112 (C01 proteases/papains); PF14541 (A01 Asp proteases/pepsins); and PF01650 (C13 legumains/VPEs). The peptides were counted per gel slice and presented as a heat map illustrating the distribution of the peptides. The outlines indicate the gel slice where the full‐length proprotease is predicted to migrate without SP but with (blue) or without (red) predicted *N*‐glycans. Proteases were ranked on their predicted MW. *Nb*TPP2 (NbL03g11790) was omitted from this analysis because it is a distinct cytoplasmic subtilase that lacks an autoinhibitory prodomain, and NbL04g13700 was omitted because it was detected by a single peptide. (b) Most peptides of detected proteases are not from the autoinhibitory prodomains. The position of all detected peptides (gray) is shown on top of the protein architectures, with the outline indicating unique (red) and non‐unique (black) peptides. (c–f) Example peptographs revealing proteoforms of two subtilases. Peptograph features are explained in Fig. 2. Catalytic residues are highlighted red within the catalytic domains. Small isoforms are highlighted with dotted ovals.

To investigate whether the PROTOMAP dataset displays known protein processing events, we mined the dataset for secreted proteases that carry an autoinhibitory prodomain that is removed upon activation, resulting in a mature protease with a lower MW. Using searches for the Pfam code of the catalytic domain for proteins detected with more than a single peptide, we identified 24 subtilisin‐like proteases (SBTs, family S08, PF00082); 16 papain‐like cysteine proteases (PLCPs, family C01, PF00112); 14 pepsin‐like aspartic proteases (family A01, PF14541); and three vacuolar processing enzymes (VPEs, family C13, PF01650). Most of these proteases accumulate at a MW that is predicted for the mature proteases and not their proenzymes (Fig. 3a), indicating that these enzymes do no longer carry their autoinhibitory prodomain. The position of the identified peptides within the protease precursors confirms this pattern: Most peptides originate from the protease domains (Fig. 3b). The absence of peptides from the prodomain in any gel slice indicates that these domains are degraded *in vivo*, presumably to avoid inhibition *in trans*.

Notable exceptions are subtilases NbL03g00730 (*Nb*SBT1.4, Fig. 3c), NbL17g25630, NbL06g14490 and NbL16g14350, for which peptides were detected of the prodomain, but these peptides were only detected at 15 kDa rather than the MW of the subtilase domain (Notes S2), indicating that these prodomains have been removed by processing but that they persist. Likewise, instead of being detected at the MW of the proprotease, peptides for the prodomain were also detected at 15 kDa for PLCP NbL05g12610, an aleurain‐like protease (*Nb*ALP, Fig. 3d), suggesting that this prodomain also persists after release. By contrast, peptides of the prodomain were also detected for PLCPs NbL12g09520, NbL13g16620 and NbL04g14460, but these peptides all migrate at the predicted MW of these proteins (Notes S2), indicating that they are from precursor proteases. Another exception is the detection of peptides from the C‐terminal inhibitory domain of VPE NbL17g4760, which were detected both at the predicted MW and at 20 kDa (Notes S2), indicating that only part of this protease is processed but that the inhibitory domain can persist separately.

Interestingly, peptograph of subtilase NbL02g06410 (*Nb*SBT1.7, Fig. 3e) also displays C‐terminal peptides corresponding to the fibronectin type‐III domain (Fn3, PF17766) at 12 kDa, indicating that this domain is released from these subtilases. Released Fn3 domains were also detected for subtilases NbL16g14350, NbL06g14490, NbL03g00730 and NbL13g19160. By contrast, the peptograph of subtilase NbL11g14620 (*Nb*SBT1.6, Fig. 3f) displays multiple C‐terminal peptides at 35 kDa, indicating that this subtilase is processed more upstream in the protease domain, severing the catalytic Ser from the catalytic Asp and His residues (Fig. 3f). It is unclear at this stage what the consequences of these cleavages are. One distinct, large cytonuclear subtilase not included in Fig. 3 is tripeptidyl peptidase (*Nb*TPP2, NbL03g11790), which was identified at 150 kDa with > 250 peptides covering its entirety (Notes S2), consistent with the high abundance and high predicted MW (147 kDa) of this cytoplasmic subtilase.

### SCPL proteases/acyltransferases are differentially processed into functional heterodimers

**Fig. 4:**
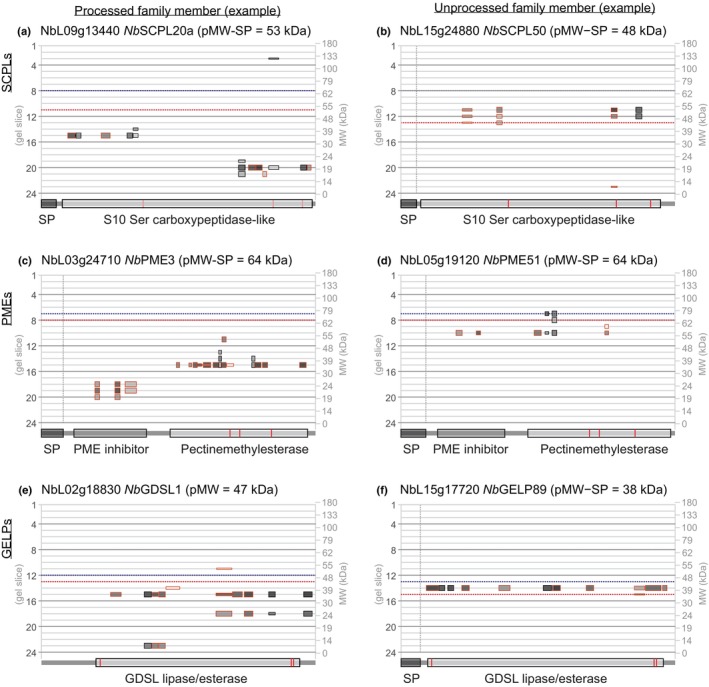
Differential processing of serine carboxypeptidase‐like (SCPLs), pectinemethylesterases (PMEs) and GDSL‐type lipase/esterase‐like proteins (GELPs). Examples of processed (a, c, e) and unprocessed (b, d, f) precursors of SCPLs, PMEs and GELPs. Catalytic residues are highlighted red within the catalytic domains. Peptograph features are explained in Fig. 2. Catalytic residues are highlighted red within the catalytic domains.

The PROTOMAP dataset contains peptographs of 18 SCPL proteins with more than one peptide, carrying catalytic domain PF00450. SCPLs can be carboxypeptidases or acyltransferases involved in secondary metabolism (Milkowski & Strack, 2004). The peptograph of *Nb*SCPL20a (NbL09g13440, see phylogeny in Fig. S4) shows that this 51 kDa enzyme is cleaved into an N‐terminal 37 kDa fragment in Slice 15, and a C‐terminal 19 kDa fragment in Slice 20 (Fig. 4a). Peptographs of 11 additional SCPLs also indicate that they accumulate as cleaved proteins (Notes S2; NbL01g02560, NbL02g23560, NbL06g01240, NbL07g00400, NbL07g17710, NbL09g11980, NbL09g13440, NbL13g00950, NbL13g01230, NbL14g14490, NbL16g05780 and NbL18g14840). These data are consistent with processing of SCPLs into a large and small subunit that function together as a disulfide‐linked heterodimer. Oat SCPL1, for instance, is cleaved into 29 and 19 kDa subunits that stay together as a disulfide‐linked heterodimer that mediate a step in the biosynthesis of antimicrobial avenacins (Mugford *et al*., 2009). Likewise, brassinosteroid insensitive‐1 suppressor BRS1 of Arabidopsis is processed into 34 and 19 kDa subunits, resulting in a heterodimer acting as a secreted carboxypeptidase (Zhou & Li, 2005). It is unclear at this stage whether SCPL processing is required for their activity, and which endopeptidase cleaves these SCPLs.

By contrast, peptides from *Nb*SCPL50 (NbL15g24880, see phylogeny in Fig. S4) are mostly in gel Slice 11 at 50 kDa, consistent with an uncleaved predicted MW of 48 kDa (Fig. 4b). Peptographs of five additional SCPLs show peptides mostly at their predicted MW, indicating that these SCPLs are not processed (Notes S2; NbL01g18230, NbL02g04500, NbL04g07300, NbL05g22050, NbL07g00690, NbL12g02780, NbL14g17630 and NbL15g24880). The differential processing of SCPLs is thought to be caused by the length and sequence of the linker peptide located between the two subunits in the SCPL sequence (Mugford *et al*., 2009). Shorter linker peptides are generally not processed. Indeed, alignment of the 18 detected SCPLs, ranked on the length of the linker region, reveals that uncleaved SCPLs have a short linker region (4–15 residues), in contrast to cleaved SCPLs that have longer linker regions (32–65 residues, Fig. S5). The alignment also shows that these cleaved SCPLs have highly polymorphic linker sequences, although many carry dibasic motifs that might be targeted by subtilases that have furin‐like activities (Fig. S5).

### PMEs are differentially processed to remove the inhibitor domain

The PROTOMAP dataset contains 12 type‐I pectinemethylesterases (PMEs) with two or more peptides. Type‐I PMEs consist of a signal peptide for secretion, followed by a 20 kDa inhibitory domain (PF04043) and the 35 kDa PME domain (PF01095). Eight of the detected PMEs migrate in protein gels as processed, mature enzymes at 35–45 kDa. *Nb*PME3 (NbL03g24710, see phylogeny in Fig. S6), for instance, shows peptides of the catalytic domain at 40 kDa and peptides of the inhibitory domain at 20 kDa (Fig. 4c). In contrast to the eight processed PMEs, the other four PMEs are detected close to the predicted MW. For instance, peptides from both the inhibitory and catalytic domains of *Nb*PME51 (NbL05g19120) are detected at 60 kDa, close to the predicted MW of 62 kDa, confirming that this PME accumulates as an uncleaved precursor (Fig. 4d).

The removal of the inhibitory domain is required to activate and secrete PMEs, which then regulate the direction of cell growth by targeted cell wall relaxation (Wolf *et al*., 2009). A dibasic motif (e.g. RRLL) at the junction between the inhibitory and catalytic domains is required for cleavage (Wolf *et al*., 2009), and Arabidopsis SBT3.3 was found to be responsible for processing PME17 (Sénéchal *et al*., 2014). Interestingly, all eight processed PMEs detected by PROTOMAP contain either RRLL (4×), RKLL (2×), RVLL or RTLL at the putative cleavage region, whilst four unprocessed PMEs lack basic residues in this region with the exception of *Nb*PME40 (NbL01g19610), which carries RALL (Fig. S7).

### GELPs are differentially processed, separating its catalytic triad

The PROTOMAP dataset contains 33 GDSL esterases/lipases (GELPs, PF00657, see phylogeny in Fig. S8), with two or more peptides. GELPs are lipolytic enzymes that contain a 35 kDa catalytic domain that contains the catalytic Ser in an N‐terminal GDSL motif (Shen *et al*., 2022). Peptographs of eight GELPs (e.g. NbL02g18830, *Nb*GDSL1, Fig. 4e) show additional peptides at 25 kDa from the C‐terminal half and peptides at 13 kDa from the N‐terminal half of the catalytic domain. This indicates that these GELPs are processed into two subunits that are stable, possibly by remaining in a heterodimeric state. Interestingly, such processing would separate the catalytic triad, which consists of the catalytic Ser residue in the N terminal domain, and the catalytic Asp and His residues in the C terminal domain (Fig. 4e). Although several plant GELPs have been biochemically characterized (e.g. Zhang *et al*., 2017; Tang *et al*., 2020), their processing *in planta* has not been reported before. By contrast, the other 25 detected GELPs only show peptides at the predicted MW from the entire catalytic domain (e.g. NbL15g17720, *Nb*GELP89, Fig. 4f), indicating that they are not processed. The mechanisms and consequences underlying this differential processing remain to be identified.

### Glycosidases are often cleaved from their C‐terminal domains

**Fig. 5:**
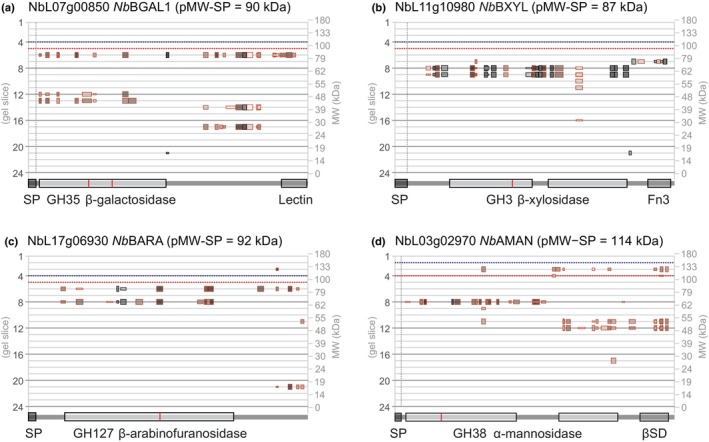
Glycosyl hydrolases (glycosidases) have multiple processing isoforms. Shown peptographs are from: (a) β‐galactosidase‐1 *Nb*BGAL1; (b) putative β‐xylosidase *Nb*BXYL; (c) putative β‐arabinofuranosidase *Nb*BARA; and (d) putative α‐mannosidase *Nb*AMAN. Peptograph features are explained in Fig. 2. Catalytic residues are highlighted red within the catalytic domains.

The PROTOMAP dataset contains peptographs of 96 glycosyl hydrolases (GHs, also called glycosidases) that mostly act in the endomembrane system and apoplast to modify the glycans in the cell wall, membrane surface and proteins (Léonard *et al*., 2009). Many GHs carry carbohydrate‐binding modules (CBM) that enable substrate selection and localization. Interestingly, the peptographs of GHs often indicate that the CBM or other C‐terminal domains are processed and can be detected at a lower MW, indicating that it is not degraded. β‐galactosidase‐1 (*Nb*BGAL1, NbL07g00850, family GH35), for instance, carries a galactose‐binding lectin domain (PF02140) at the C terminus, which is detected at 15, 28 and 40 kDa in the *Nb*BGAL1 peptograph (Fig. 5a). Peptides from the catalytic domain (PF01301) of *Nb*BGAL1 migrate at the predicted MW (90 kDa) and at 48 kDa, consistent with activity‐based labeling of this proteoform in our previous experiments (Chandrasekar *et al*., 2014; Buscaill *et al*., 2019). The four other detected BGALs of family GH35 (NbL03g01030, NbL02g14360, NbL13g19810 and NbL03g00970) all have similar peptographs, indicating that the processing of the C‐terminal lectin domain is common for the GH35 family.

Likewise, putative β‐xylosidase *Nb*BXYL (NbL11g10980, family GH3) carries a C‐terminal fibronectin III‐like domain (Fn3, PF14310) that can be detected at 75 kDa as part of the full‐length protein (Fig. 5b). In addition to the full‐length protein, peptides of the catalytic domain are also detected at 60 kDa, but this gel slice lacks peptides of the Fn3 domain, indicating that this proteoform is C‐terminally truncated. Although no peptides of the Fn3 domain can be detected for the four other GH3‐family β‐xylosidase carrying an Fn3 domain (NbL11g20540, NbL04g10020, NbL15g17830 and NbL01g14480), they all show peptides from only the catalytic domain migrating at 60–65 kDa, below the predicted MW, indicating that the C‐terminal Fn3 domain has been removed from all these GH3 β‐xylosidases. The function of the Fn3 domain in GH3 β‐xylosidase is unknown, but it might be involved in anchoring the enzyme on large polymeric substrates and in thermostability (Pozzo *et al*., 2010). Interestingly, we detected the processing of a different Fn3 domain (PF17766) from the C terminus of three subtilases (see subtilase section).

Likewise, putative β‐arabinofuranosidase *Nb*BARA (NbL17g06930, family GH127) is detected at 88 kDa, close to its predicted MW of 92 kDa, but the catalytic GH127 domain also migrates at 65 kDa. This shorter proteoform lacks peptides from the C‐terminal region, whilst this C‐terminal region is detected at 15 kDa (Fig. 5c). This indicates that the C‐terminal region is removed from this GH127 glycosidase. The C‐terminal region has homology to two β‐sandwich domains that are important for homodimerization of a bacterial GH127 enzyme (Ito *et al*., 2014), but it is unknown how this processing might influence GH127 function.

Several other glycosidases show interesting processing events. The peptograph of heparanase‐like *Nb*HEPA (NbL13g02440, family GH79), for instance, has peptides covering most of the protein at 58 kDa, consistent with its predicted MW (56 kDa), but also peptides of the catalytic domain (PF03662) at 35 kDa and even a smaller fragment at 15 kDa (Notes S2), indicating that this glycosidase is processed. In addition, the peptograph of putative α‐mannosidase *Nb*AMAN (NbL03g02970, family GH38) shows peptides across the protein at 130 kDa, close to its predicted MW (114 kDa), but much more and intense peptides of the N‐terminal GH38 domain (PF01074) at 65 kDa and peptides of the C‐terminal GH38 domain (PF07748) at 50 kDa (Fig. 5d), indicating that most of this hydrolase is cleaved into two subunits. This peptograph is consistent with the observation that a homologous GH38 α‐mannosidase from tomato fruits contains 70 and 47 kDa subunits (Hossain *et al*., 2009).

### Transient expression of double‐tagged precursors confirms processing *in vivo*

**Fig. 6:**
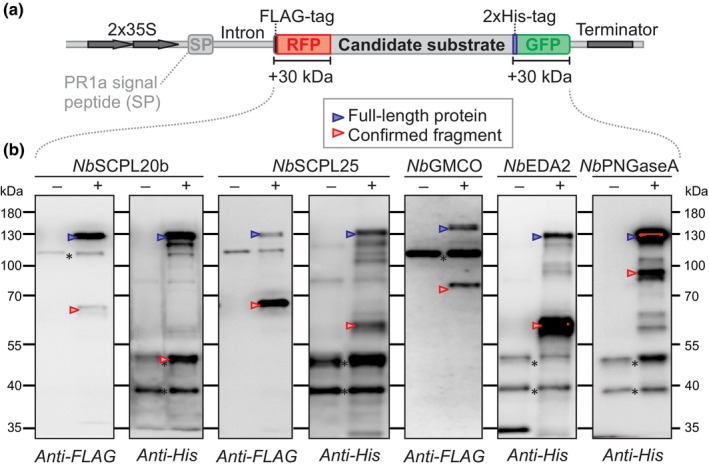
Several processing events are confirmed upon transient expression. (a) The coding region of candidate proteins without their own signal peptide were cloned in‐between sequences encoding SP‐FLAG‐RFP and 2xHis‐GFP. These coding sequences are located on the T‐DNA of binary vectors and are driven by a 2 × 35S promoter and terminated by a 35S terminator and carry an intron to avoid protein expression by bacteria. (b) Western blots of double‐tagged proteins confirm several processing events *in vivo*. *Nicotiana benthamiana* leaves were agroinfiltrated with the empty vector (−) or the double‐tagged candidate proteins (+) and extracted in gel loading buffer for Western blot analysis with anti‐FLAG and anti‐His antibodies to detect the N‐ and C‐termini of the fusion proteins, respectively. *, background signal, also seen in the empty vector control.

To confirm the existence of proteoforms of secreted proteins *in vivo*, we transiently expressed several extracellular proteins with suspected processing by agroinfiltration with tags on both sides of the protein (Fig. 6a). We added an N‐terminal RFP and a C‐terminal GFP protein to detect expression before protein extraction and to ensure that also processing near the N‐ and C‐terminus can be detected by Western blots. We also added an N‐terminal FLAG tag and C‐terminal His tag because we found that antibodies against these tags are more sensitive than anti‐RFP/GFP antibodies. The double‐tagged protein was fused behind the signal peptide of PR1a to ensure entry into the secretory pathway. These double‐tagged constructs were driven by the 2x35S promotor and contain an intron to exclude bacterial expression.

We first selected two SCPLs that are processed according to the PROTOMAP dataset. Peptides of *Nb*SCPL20b (NbL09g13440, see phylogeny in Fig. S4) were detected at 37 kDa (N‐terminal part) and 20 kDa (C‐terminal part) (Fig. S9A). Both fragments were confirmed with double‐tagged SCPL20b, with tagged fragments accumulating at 65 and 45 kDa, respectively (Fig. 6b). Likewise, the peptograph of *Nb*SCPL25 (NbL02g23560) displayed peptides at 40, 30 and 25 kDa, indicating full‐length, N‐ and C‐terminal fragments, respectively (Fig. S9B). Western blot analyses of double‐tagged SCPL25 confirmed the accumulation of both the tagged N‐ and C‐terminal fragments, accumulating at 65 and 55 kDa, respectively (Fig. 6b). These data are consistent with the processing of SCPLs as explained previously.

The peptograph of glucose‐methanol‐choline oxidoreductase *Nb*GMCO (NbL10g21430) shows peptides at 64 kDa, representing the predicted full‐length protein lacking its signal peptide, but also at 50 kDa, with peptides from the N‐terminal half, and at 28 kDa with peptides from the C‐terminal half (Fig. S9C). This indicates that *Nb*GMCO is cleaved between the N‐terminal FAD‐binding domain and the substrate binding domain. To confirm this observation, we double‐tagged GMCO and monitored its accumulation upon transient expression by agroinfiltration. Western blot analysis confirmed the accumulation of the N‐terminal processing product, accumulating at 75 kDa (Fig. 6b), but the C‐terminal processing product could not be detected, possibly because of another processing event at the junction with the 2xHis‐GFP reporter tag.

The peptograph of the putative serine protease *Nb*EDA2 (NbL06g09140, S28 family, PF05577) shows most peptides at 53 kDa, consistent with its predicted MW (52 kDa), but also shows N‐terminal peptides at 28 kDa and C‐terminal peptides at 25 kDa (Fig. S9D). Western blot analysis of transiently expressed double‐tagged *Nb*EDA2 confirmed the accumulation of the His‐GFP‐tagged C‐terminal fragment at 60 kDa (Fig. 6b), whereas the N‐terminal fragment was not detected. Interestingly, this cleavage would separate the catalytic triad since the catalytic Ser residues locates in the N‐terminal half, and the catalytic Asp and His residues at the C terminus. However, as with SCPLs, this cleavage might result in a functional heterodimer.

Finally, the peptograph of putative Golgi‐localized peptide‐N4‐(N‐acetyl‐beta‐glucosaminyl) asparagine amidase A (*Nb*PNGaseA, NbL17g03090) shows peptides of the majority of the protein at 58 kDa, which might be the full‐length protein (62 kDa), and both C‐ and N‐terminal peptides at 45 kDa, indicating that this protein is processed (Fig. S9E). Western blot analysis of transiently expressed double‐tagged *Nb*PNGaseA confirmed the accumulation of a His‐GFP‐tagged C‐terminal fragment at 80 kDa, consistent with the peptograph (Fig. 6b). Taken together, these Western blot experiments confirmed that the cleavages observed in the peptographs occur *in vivo* and can be detected by transient expression of double‐tagged precursor proteins.

### Detection of extracellular domains of RLKs indicates ectodomain shedding

Interestingly, the PROTOMAP dataset also contains peptides of receptor‐like kinases (RLKs). This was unexpected because the sample preparation included the removal of insoluble material by centrifugation before boiling in lithium dodecyl sulfate. To investigate this further, we searched the data for RLKs using Pfam identifiers for the protein kinase domain (PF00069 and PF07714) preceded by a signal peptide and/or transmembrane domain. This search delivered 37 putative RLKs, each with at least two peptides, including likely orthologs of the brassinosteroid receptor BRI1/SR160 (Wang *et al*., 2018); chitin receptor CERK1 (Miya *et al*., 2007); RALF1 receptor FERONIA (Haruta *et al*., 2014); cell wall sensor HERK1 (Guo *et al*., 2009); BAK1‐regulator BIR2 (Halter *et al*., 2014); and auxin co‐receptor TMK1 (Friml *et al*., 2022).

**Fig. 7:**
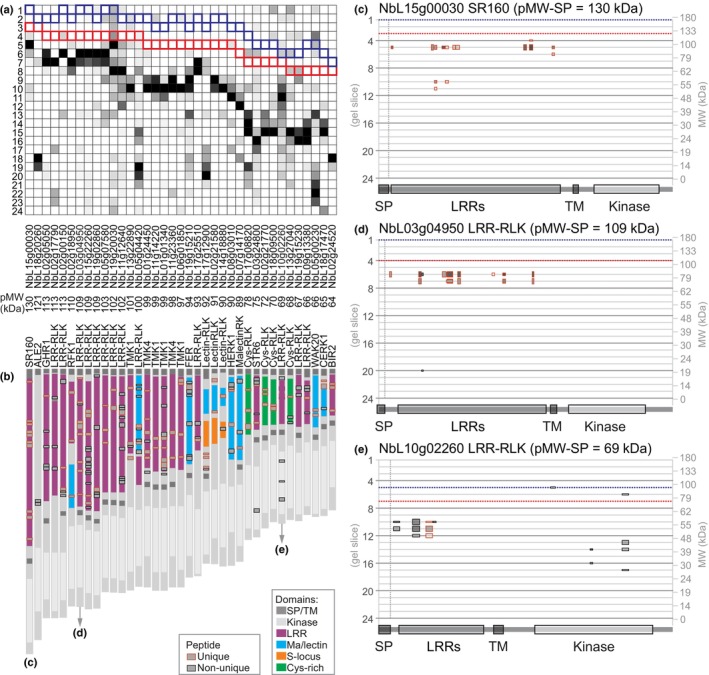
Detection of processed ectodomains suggests frequent receptor shedding in plants. (a) Peptides from receptor‐like kinases (RLKs) migrate below their predicted molecular weight (MW). Peptides detected from RLKs are summarized for each gel slice, with highest coverage and ion intensity (black), medium ion intensity (dark gray) and low ion intensities (light gray). RLKs were extracted by selecting peptographs that contain protein kinase domains PF07714 or PF00069, preceded by a signal peptide (SP, not shown) and/or transmembrane domain (TM). RLKs identified by a single peptide were omitted. RLKs were ranked on their predicted MW. Outlined is the gel slice where the full‐length RLK is predicted to migrate without SP but including predicted *N*‐glycans (blue line) or without predicted *N*‐glycans (red line). (b) Peptides of detected TM receptors are almost exclusively from the ectodomains. The position of the detected peptides is shown as gray boxes on top of the domain architecture as unique (red outline) or non‐unique (black outline). (c–e) Example peptographs of processed RLKs. Peptograph features are explained in Fig. 2.

Notably, most RLK‐derived peptides were found in gel slices much below the gel slice that is predicted to contain full‐length, *N*‐glycosylated RLKs (Fig. 7a), indicating that these RLKs are truncated. This truncation is consistently 50–70 kDa, irrespective of the type of RLK. Further inspection of the detected peptides revealed that nearly all RLK‐derived peptides originate from ectodomains and only few peptides are from kinase domains, despite being a substantial part of the RLK protein (Fig. 7b). Putative brassinosteroid receptor BRI1/SR160 (NbL15g00030), for instance, is detected by peptides exclusively from the ectodomain at 90 kDa, 100 kDa below its predicted MW when fully *N*‐glycosylated (Fig. 7c). Likewise, LRR‐RLK NbL03g04950 is detected with only ectodomain peptides at 80 kDa, 77 kDa below its predicted MW when fully *N*‐glycosylated (Fig. 7d). Taken together, these data indicate that we detected a wide range of ectodomains that were cleaved from the membrane, releasing a soluble extracellular domain. The remaining TM‐kinase domains probably remained in the membrane fraction that was pelleted during sample preparation. These data suggest that ectodomains of RLKs representing different RLK subfamilies are shed from the membrane.

A notable exception is LRR‐RLK NbL10g02260 (Fig. 7e), which has a predicted MW of 90 kDa including *N*‐glycans, but for which peptides derived from the kinase domain were detected at 40–44 kDa, corresponding to the predicted MW of the cytoplasmic domain. The peptograph also shows peptides of the ectodomain at 55 kDa, consistent with ectodomain shedding. These data suggest that this LRR‐RLK is cleaved on both sides of the membrane, releasing both a soluble ectodomain, and a soluble kinase domain.

## Discussion

Our conceptually simple PROTOMAP experiment has uncovered 2977 endogenous plant proteins that have multiple signals in protein gels. Whilst most are novel and implicate extensive protein processing in agroinfiltrated *N. benthamiana* leaves, these processing events are consistent with the literature. Nine processing events were confirmed by Western blot analysis by transiently expressing double‐tagged proteins. The PROTOMAP dataset is an extensive source of putative endogenous protease substrates to unravel the proteolytic machinery of this protein production platform in the future.

The PROTOMAP dataset is a rich source of proteoforms in *N. benthamiana* leaves, most of which have not yet been reported before. This PROTOMAP dataset contrasts with other approaches to detect protein processing such as TAILS/HUNTER and COFRADIC at several points. First, although TAILS/HUNTER and COFRADIC will reveal cleavage sites, they do not display the MW of the protein isoform from which the peptide was generated, meaning that some of the identified neo‐termini may originate from further degradation products, unless other experiments show the accumulation of corresponding isoforms. Second, it is unclear from TAILS/HUNTER and COFRADIC datasets whether the detected processing event is a minority or majority processing event. PROTOMAP, by contrast, displays the relative abundance of the cleaved products and precursor, forcing the focus on major cleavage events that cause a shift of the protein in the gel that can be confirmed by Western blot. Third, in contrast to TAILS/HUNTER and COFRADIC, PROTOMAP does not display cleavage site information. Although we have searched these data for half‐tryptic peptides, only very few of these half‐tryptic peptides were located in the putative cleavage regions. We did not include the detection of half‐tryptic peptides in the current dataset because these searches expand the search space and reduce the confidence of the detected tryptic peptides of the PROTOMAP dataset.

The PROTOMAP dataset is an extensive source of proteoforms that can be explored further for protein processing and abundance in agroinfiltrated *N. benthamiana* leaves. We mostly focused our further analysis on secreted proteins and have highlighted several phenomena in this dataset, which are to be briefly discussed later.

### Zymogen maturation

PROTOMAP analysis uncovered numerous processing events that are consistent with the literature. For instance, the prodomain of proteases was found to be removed in our dataset for papains, subtilases, legumains and pepsins. It is interesting to note that we did not detect peptides of most of the prodomains, indicating that they are degraded as soon as they are released from the precursor protein. The differential stability of different parts of secreted proteins suggests mechanisms in extracellular protein homeostasis that are not yet understood.

### Differential processing within protein families

We also detected differential processing within the SCPL, PME and GELP protein families. Processing of these proteins is consistent with the literature on family members. In SCPLs, cleavage occurs in a polymorphic linker region and cleaved proteins continue to function as heterodimers (Mugford *et al*., 2009). The PMEs require processing at a dibasic RKxL/RRxL‐like motif to remove the autoinhibitory prodomain (Wolf *et al*., 2009). Processing of GELPs separate their catalytic triad but the cleavage site is unknown, and it is unknown whether this affects the function of these enzymes.

### Processing of glycosidases

We also discovered that glycosidases are processed. We detected the separation from the C‐terminal domain in three different glycosidase families, and we highlighted the processing of glycosidases of the GH79 and GH38 families. It is unclear how these processing events affect glycosidase function, but the removal of CBDs is likely to affect the localization and specificity of these enzymes.

### RLK processing

Interestingly, the PROTOMAP dataset contains peptides of 37 RLKs suggesting receptor processing. Processing leads to the regulation of the activity of receptors via activation or deactivation but also to the generation of proteoforms with novel functions (Lichtenthaler *et al*., 2018). In most cases, peptides from ectodomains but not of the kinase domains were found. These data are consistent with ectodomain shedding that has been reported widely in mammals (Lichtenthaler *et al*., 2018). In plants, by contrast, only few ectodomain shedding events have been reported: Chitin receptor CERK1 in Arabidopsis is cleaved in the second LysM motif of its ectodomain, generating a N‐terminal 33 kDa fragment. The *cerk1‐4* mutant harbors a point mutation that impedes the generation of this N‐terminal shedding product, and these mutants are still able to perceive chitin but show enhanced cell death upon biotic stress (Petutschnig *et al*., 2014). Likewise, processing in the extracellular malectin‐like domain of SYMRK from *Lotus japonicus* generates a truncated version of the receptor that still contains an extracellular LRR domain but is less stable and more prompt to form a complex with the Nod factor receptor 5 (NFR5), thereby activating symbiotic development (Antolín‐Llovera *et al*., 2014).

We also found one LRR‐RLK (NbL10g02260) that shows not only putative ectodomain shedding but also a kinase migrating at 40–44 kDa, indicating that this kinase is released from the membrane. Research on Notch receptors in mammals has shown that ectodomain shedding can induce subsequent processing and release of a cytosolic proteoform (De Strooper *et al*., 1999). In plants, the proteolytic generation of cytosolic proteoforms of RLKs has been reported in four cases (Xa21 (Park & Ronald, 2012), BAK1 (Zhou *et al*., 2019), TMK1 (Gu *et al*., 2022) and FERONIA (Chen *et al*., 2023)). Processing of the detected RLKs regulates signaling in plants is an exciting topic for future investigations.

### Double‐tagged processing reporters

We used Western blot analysis of transiently expressed double‐tagged proteins to confirm several of the observed cleavage events. We noticed that some processing events were not detected by Western blot of double‐tagged precursors. There are several reasons for this. First, all proteins also accumulated as full‐length proteins, even though these were often not detected in the peptographs. This is probably caused by the continuous overproduction of these proteins, causing either an overdose when compared to the processing enzymes, or an accumulation of the precursor before their processing. Second, not all processing events were detected with double‐tagged vectors. This could be caused by additional processing events closer to the N‐ and C‐termini resulting in the removal of the reporter tags. The bulky reporter tags may also have caused misprocessing or mislocalization of the tagged protein. We nevertheless were able to confirm processing of several proteins, testifying that these cleavages occur *in vivo* and enabling us to use these proteins in the future as reporters to identify the proteases responsible for these cleavage events.

### Further use of PROTOMAP data

Our PROTOMAP dataset can be mined further by searching the peptographs in the PDF file (Fig. S2) with identifiers, Pfam domains, sequences and functional names. A PDF editor can be used to quickly extract all peptographs with shared Pfam codes, identifiers or functional names. Extra care should be taken with the interpretation of peptographs showing very few peptides, but these peptographs are usually consistent with peptographs of other members of the same protein family. It should be stressed that the different proteoforms in peptographs may also result from alternative splicing and alternative initiation of transcription and translation. These cases can be identified using alternative gene models and mapping RNA seq reads on genome sequences. In addition, our PROTOMAP dataset lacks information of cleavage sites because it only contains tryptic peptides. Our searches for half‐tryptic peptides reduced the confidence threshold, which drastically reduced the number of tryptic peptides and did not deliver many reliable cleavage events.

In conclusion, this PROTOMAP dataset revealed many fascinating novel proteoforms of plant proteins. We discovered that 59.7% of the proteins exist in multiple proteoforms that run at different apparent MWs in protein gels and are presumably the result of protein processing by proteases *in vivo*. This includes prodomain removal, the separation of catalytic triads and receptor shedding. Processing is often consistent within protein families, but there are also interesting exceptions. These data indicate extensive protein processing pathways in agroinfiltrated *N. benthamiana* and identified substrates that will facilitate future studies on protein processing in *N. benthamiana*.

## Competing interests

None declared.

## Author contributions

RALvdH conceived and managed the project; ELT produced the protomap samples; KZ mined the dataset to identify processing events and confirmed processing with double‐tagged precursors; SN, FK and MK generated proteomics data; ELT and JCL analyzed MS data; NS supported PROTOMAP analysis and build phylogenetic trees; MS analyzed receptor processing; RALvdH wrote the article with input from all co‐authors.

## Supporting information


**Fig. S1** Image of 4–12% gradient SDS‐PAGE used for PROTOMAP.
**Fig. S2** Number of annotated (unique) tryptic peptides to LAB3.60 vs NbDE proteomes.
**Fig. S3** Correlation between predicted and apparent molecular weight for proteins detected with a single peptide.
**Fig. S4** Phylogenetic tree of Arabidopsis and *Nicotiana benthamiana* serine carboxypeptidase‐like.
**Fig. S5** Alignment of the linker region of serine carboxypeptidase‐like.
**Fig. S6** Phylogenetic tree of Arabidopsis and *Nicotiana benthamiana* pectin methylesterase.
**Fig. S7** Alignment of the linker region of pectin methylesterase.
**Fig. S8** Phylogenetic tree of Arabidopsis and *Nicotiana benthamiana* GDSL‐like lipase/acylhydrolase.
**Fig. S9** Peptographs of five processed proteins confirmed by double tagging.

**Notes S1** All detected peptides.

**Notes S2** Peptographs of all 5040 proteins.

**Notes S3** Protomap scripts.

**Notes S4** Uncropped images.
**Table S1** Used plasmids.
**Table S2** Used oligonucleotides.
Please note: Wiley is not responsible for the content or functionality of any Supporting Information supplied by the authors. Any queries (other than missing material) should be directed to the *New Phytologist* Central Office.

## Data Availability

The peptide file that was used as an input for the PROTOMAP is provided as Notes S1. The code that was used to generate the PROTOMAP is in Notes S3 and available on GitHub (https://github.com/joyylyu/protomap.git). Uncropped images are provided in Notes S4.
