# Supplementary material for: The proteome of Nicotiana benthamiana is shaped by extensive protein processing
Source: New Phytol. 2024 Jun 9;243(3):1034–49. doi: 10.1111/nph.19891 (PMC11494411; doi:10.1111/nph.19891)
Supplement: Supplementary file 1 — Fig. S1 Image of 4–12% gradient SDS‐PAGE used for PROTOMAP. Fig. S2 Number of annotated (unique) tryptic peptides to LAB3.60 vs NbDE proteomes. Fig. S3 Correlation between predicted and apparent molecular weight for proteins detected with a single peptide. Fig. S4 Phylogenetic tree of Arabidopsis and Nicotiana benthamiana serine carboxypeptidase‐like. Fig. S5 Alignment of the linker region of serine carboxypeptidase‐like. Fig. S6 Phylogenetic tree of Arabidopsis and Nicotiana benthamiana pectin methylesterase. Fig. S7 Alignment of the linker region of pectin methylesterase. Fig. S8 Phylogenetic tree of Arabidopsis and Nicotiana benthamiana GDSL‐like lipase/acylhydrolase. Fig. S9 Peptographs of five processed proteins confirmed by double tagging. [file NPH-243-1034-s004.pdf]

New Phytologist Supporting Information

Article title: *The proteome of Nicotiana benthamiana is shaped by extensive protein processing*

Authors: Kaijie Zheng, Joy C. Lyu, Emma L. Thomas, Mariana Schuster, Nattapong Sanguankiattichai, Sabrina Ninck, Farnusch Kaschani, Markus Kaiser, Renier A. L. van der Hoorn

Article acceptance date: 29 April 2024

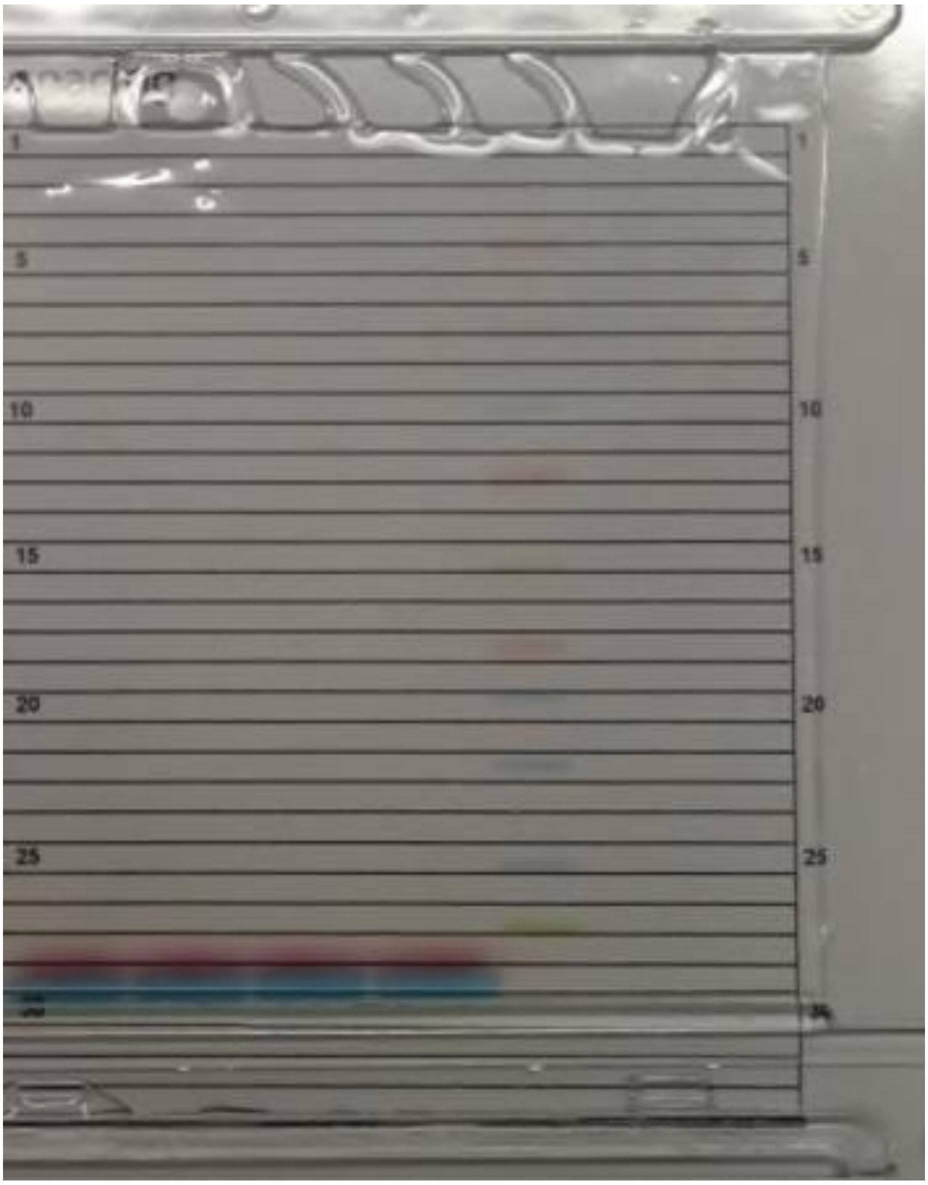

Figure S1 Image of 4-12% gradient SDS PAGE used for PROTOMAP.

Gel slices were excised in 2.5 mm bands guided by gridlines in one cut. Next, the four biological replicate lanes were separated. The prestained MW marker is just visible.

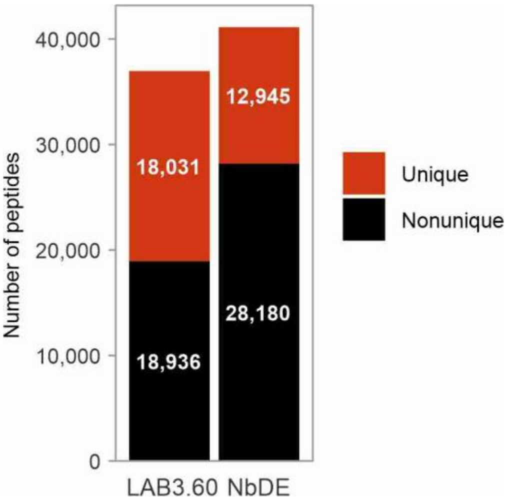

Figure S2 Number of annotated (unique) tryptic peptides to LAB3.60 vs NbDE proteomes. Spectra were annotated to the tryptic proteomes of the LAB3.60 and NbDE annotations and the number of non-unique and unique peptides were counted.

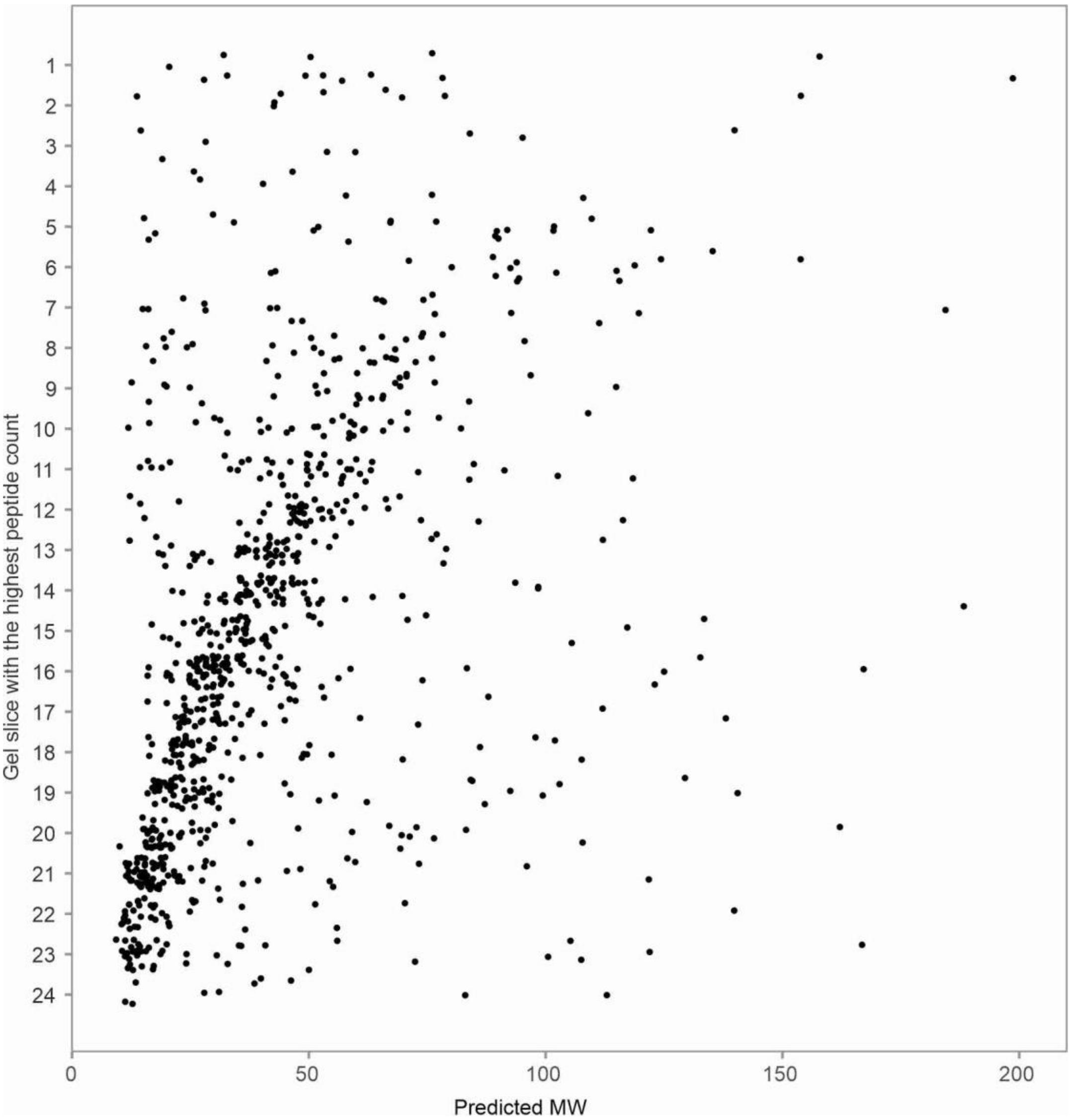

Figure S3 Correlation between predicted and apparent MW for proteins detected with a single peptide. Slices with the highest count were taken if a single peptide was detected in multiple slices.

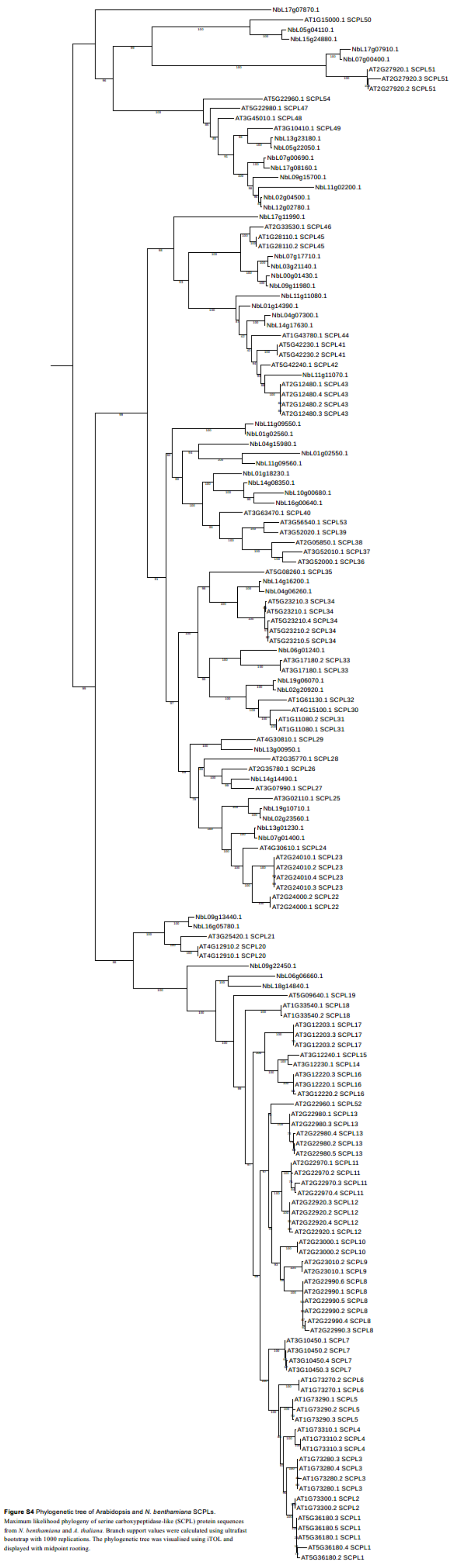

Figure S4 Phylogenetic tree of Arabidopsis and *N. benthamiana* SCPLs.

Maximum likelihood phylogeny of serine carboxypeptidase-like (SCPL) protein sequences from *N. benthamiana* and *A. thaliana*. Branch support values were calculated using ultrafast bootstrap with 1000 replications. The phylogenetic tree was visualised using iTOL and displayed with midpoint rooting.

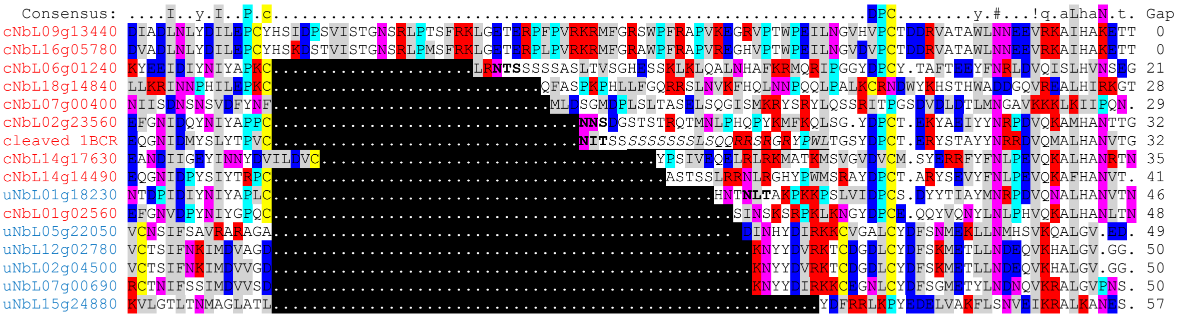

Figure S5 Alignment of the linker region of SCPLs. Shown are nine processed SCPLs (red annotation) including wheat SCPL1 (1BCR) and six unprocessed SCPLs (blue annotation). Also indicated are the sequons (NxS/T, bold) and the linker peptide that is removed from wheat SCPL1 (1bcr, underlined italics). SCPLs are ranked based on the length of the linker region.

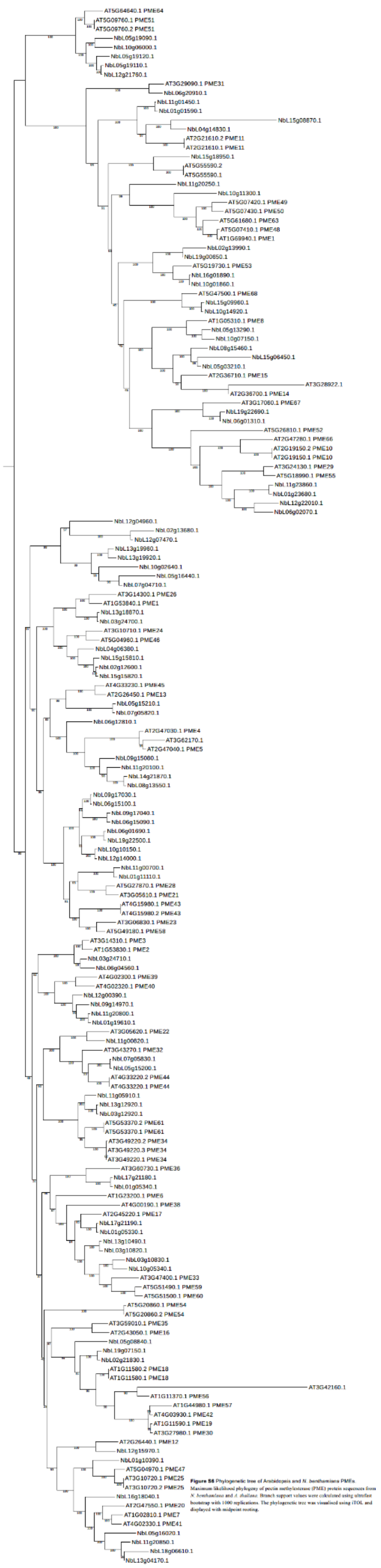

Figure S6 Phylogenetic tree of Arabidopsis and *N. benthamiana* PMEs.

Maximum likelihood phylogeny of pectin methylesterase (PME) protein sequences from *N. benthamiana* and *A. thaliana*. Branch support values were calculated using ultrafast bootstrap with 1000 replications. The phylogenetic tree was visualised using iTOL and displayed with midpoint rooting.

```
cNbL12g00390 .....YPKWLKKKDRTLLQ.APINQIKFDLVVAKDGSGNFRTINEALSTAPNSS.STRFV
cNbL03g24710 .....WPEWLSAGDRRLLQ.S..STVRPDVVVAADGSGNFKTVSEAVARAPEKS.SKRYV
cNbL13g12920 D....FPKWLSRRDRKLLN.KSVSTIQADIIVAKDGSGTVKTIAEAIKKVPEKS.NRRTI
cNbL11g05910 EDFAPFPKWLNRKERVLLD.TPVSAIHADIIVSKDGNGTFKTIAEAIKKVPQYS.NRRII
cNbL05g08840 .....MPSWVNSRDRKLLE.SPAEDIKANAVVAQDGSGDYQTLTEAVAAAPDKS.KTRYV
cNbL02g21830 .....LPTWV...DRRLLQ.LSANAIKANVIVAKDGSGQYKTVKEAVASAPDNS.KTRYV
cNbL03g24700 .....YPEWVRPGDRRLLQ.A..VNPKPDVTVASDGTRDVLTIQEAVKRVPKKS.KVRFV
cNbL05g15200 .....FPFWLNRKDRRLLQLTPSTGVVADVVVALDGTGNFTRIKDAISAAPQLS.TKRFV
uNbL05g19120 .....WEGSGSDGPCHDIKGGVPSGLKPDVTVCKEGGCDYKIVQEAVNAAPDNGLTRKFV
uNbL12g21760 .....WEGSGS.GESGQAKVGVPSGLKPDVTVCKEGGCNYKTVQEAVNAAPDNEVTRKFV
uNbL05g19090 .....WEPGSVSG..FEFKGGFPSGLDPNVTVCKEGGCDYKMVQEAVNATPDNLGPGKFV
uNbL01g19610 .....FPKWLKKRDRALLQ.ATINETEIDLVVAKDGSGNFSTINEALSAAPNAS.RTRFV
```

Figure S7 Alignment of the linker region of PMEs. Shown are eight cleaved PMEs (c, red annotation) and four uncleaved PMEs (u, blue annotation). The putative cleavage motif is highlighted in red.

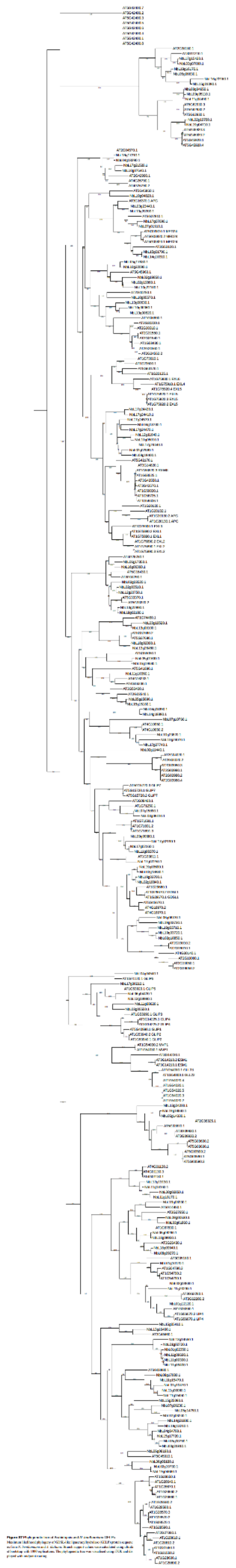

Figure S8 Phylogenetic tree of Arabidopsis and *N. benthamiana* GELPs.

Maximum likelihood phylogeny of GDSL-like lipase/acylhydrolase (GELP) protein sequences from *N. benthamiana* and *A. thaliana*. Branch support values were calculated using ultrafast bootstrap with 1000 replications. The phylogenetic tree was visualised using iTOL and displayed with midpoint rooting.

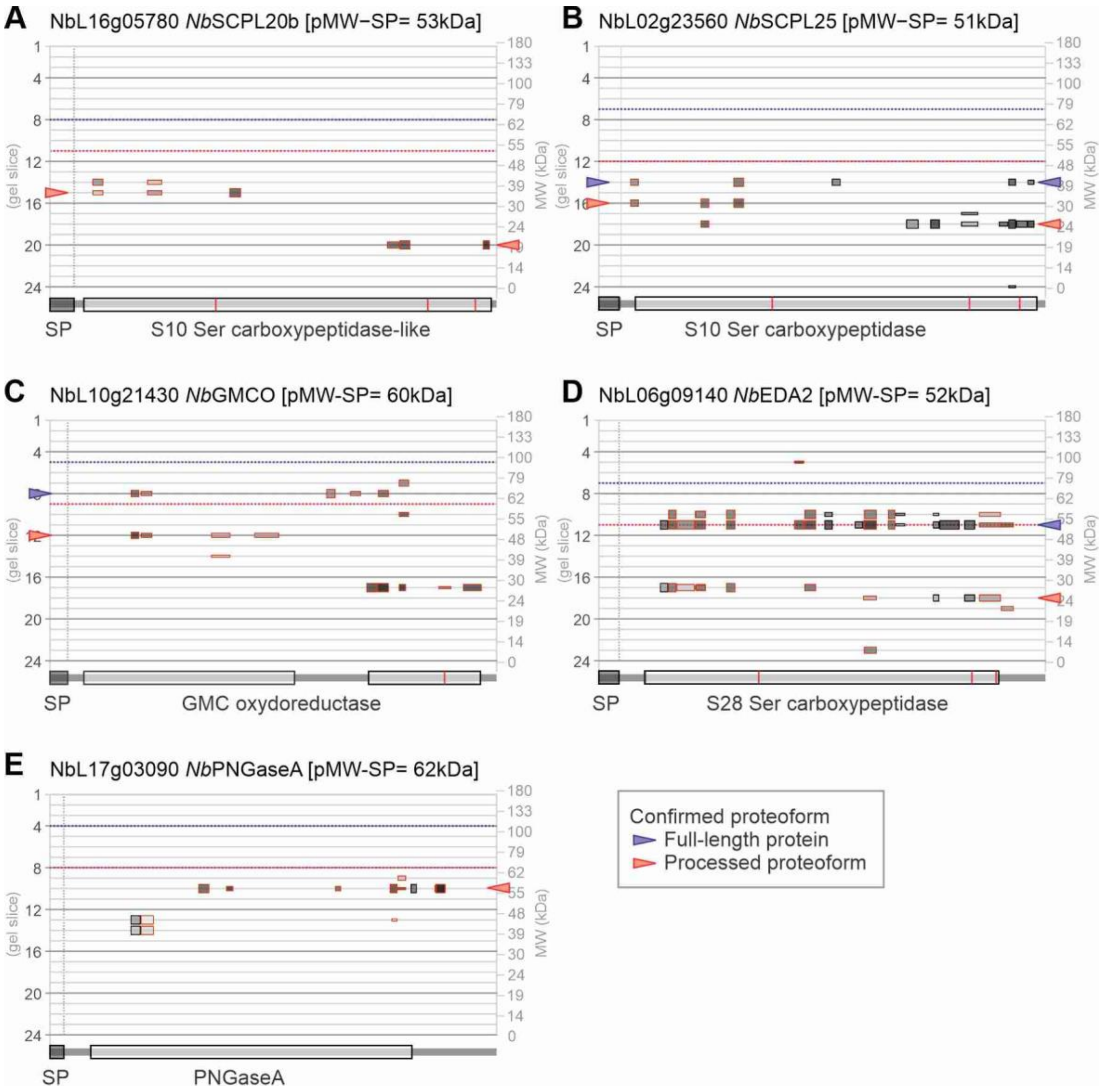

Figure S9 Peptographs of five processed proteins confirmed by double tagging.

Highlighted with arrowheads are the proteoforms that were detected *in vivo* with double-tagged constructs.

Table S1 Used plasmids

Plasmid	Description*	Reference
pJK001c	Backbone for pKZ45	Paulus et al., 2020
pJK497	Binary empty vector	Kourelis et al., 2020
pJK497	Template for 2x35S-SP-intron	Kourelis et al., 2020
pPJ057	Template for FLAG-RFP	Beritza et al., in prep.
pJK227	Template for GFP-terminator	Kourelis et al., 2020
pKZ45	Binary 35S: SP-FR-HG	This work
pKZ47	Binary 35S: SP-FR-SCPL25-HG	This work
pKZ49	Binary 35S: SP-FR-EDA2-HG	This work
pKZ54	Binary 35S: SP-FR-GMC-HG	This work
pKZ60	Binary 35s: SP-FR-PNGaseA-HG	This work
pKZ67	Binary 35S: SP-FR-SCPL20b-HG	This work

*, SP, PR1a Signal Peptide; FR, FLAG-RFP; HG, 2xHis-GFP.

Kourelis J, Malik S, Mattinson O, Krauter S, Kahlon PS, Paulus JK, van der Hoorn RAL. (2020) Evolution of a guarded decoy protease and its receptor in solanaceous plants. *Nat Commun.* **11**, 4393.

Paulus JK, Kourelis J, Ramasubramanian S, Homma F, Godson A, Hörger AC, Hong TN, Krahn D, Ossorio Carballo L, Wang S, Win J, Smoker M, Kamoun S, Dong S, van der Hoorn RAL. (2020) Extracellular proteolytic cascade in tomato activates immune protease Rcr3. *Proc. Natl. Acad. Sci. USA.* **117**, 17409-17417.

Table S2 Used oligonucleotides

Name	Sequence (5' – 3')
SPintron-EcoRI-F1	CAAgaattcGGATCCGGAGGTCAACATGGTGGAGCAC
2x35S-SPi-SalI-R1	CAAgtcgacCTGCACATCAACAAATTTTGGTCATATATTAG
FLAGRFP-SalI-F1	CAAgtcgacGAATGGATTACAAGGATGACGACGATAAGGAGCCGCGGATGGCCT CCTCCGAGGAC
FLAGRFP-KpnI-R1	CAAggtaccACTAGTCTTAAGGGCGCCGGTGGAGTGGCGGCC
HisGFPterm-KpnI-F1	CAAggtaccCCCGGGTCTAGAACGCGTCATCACCATCACCATCACCATCACCATC ACCATCACCTGCAGATGGTGAGCAAGGGCGAGG
HisGFPterm-PvuI-R2	CAAcgatcgACTCTAGCTAGAGAAGCTGATCAATGCATC
SCPL25-AflII-F1	CAActtaagACCAATTACAAGGAAGAAGAAGAAGCTG
SCPL25-XbaI-R1	CAAtctagaTGACTTGGGAAGTGGCTCTC
EDA2-KpnI-F1	CAAggtaccATCTCAACTTCTCATCTTCTTCTTCAG
EDA2-XbaI-R1	CAAtctagaTACATCTGAAACCTGGCACTG
GMC-AflII-F2	CAActtaagGAAAAAGCTCCAAACTACTCATTC
GMC-KpnI-R1	CAAggtaccATTAATATGACTCTTCTCTTTTGCAAG
PNGaseA-AflII-F1	CAActtaagCTTGAACACCATTCCCACTTC
PNGaseA-KpnI-R1	CAAggtaccCAAAGCAGAAAACGAATCAAACTTC
SCPL20b-AflII-F1	CAActtaagGTACCTGAAAATGCATTAATTACTCAAATTCCTG
SCPL20b-XbaI-R1	CAAtctagaTATGTTCTTGCCCTCTAGCCAG

Restrictions sites for restriction enzymes indicated in the primer name are printed in small case.
