# Supplementary material for: The proteome of Nicotiana benthamiana is shaped by extensive protein processing
Source: New Phytol. 2024 Jun 9;243(3):1034–49. doi: 10.1111/nph.19891 (PMC11494411; doi:10.1111/nph.19891)
Supplement: Supplementary file 5 — Notes S4 Uncropped images. Table S1 Used plasmids. Table S2 Used oligonucleotides. Please note: Wiley is not responsible for the content or functionality of any Supporting Information supplied by the authors. Any queries (other than missing material) should be directed to the New Phytologist Central Office. [file NPH-243-1034-s001.pdf]

3 x (EV; FLAG-RFP-*Nb*SCPL25-His-GFP)

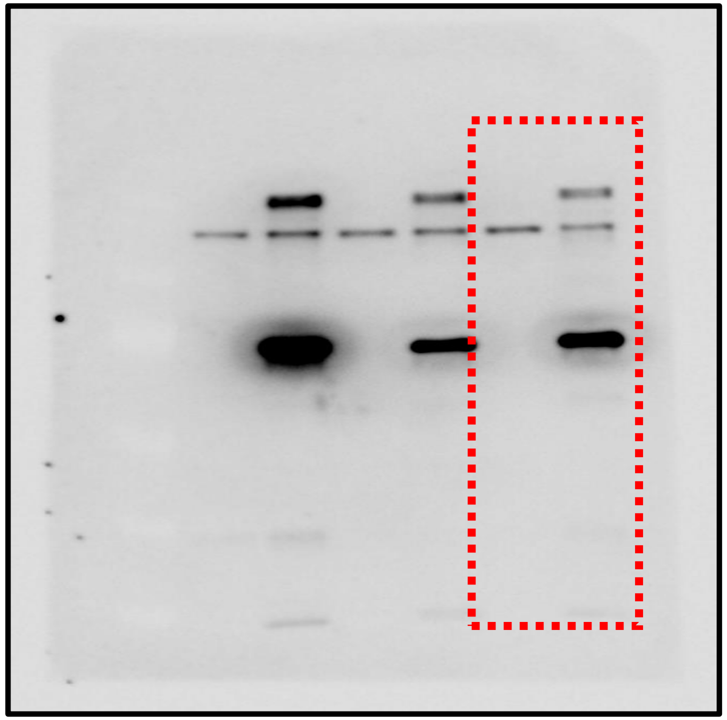

Anti-FLAG

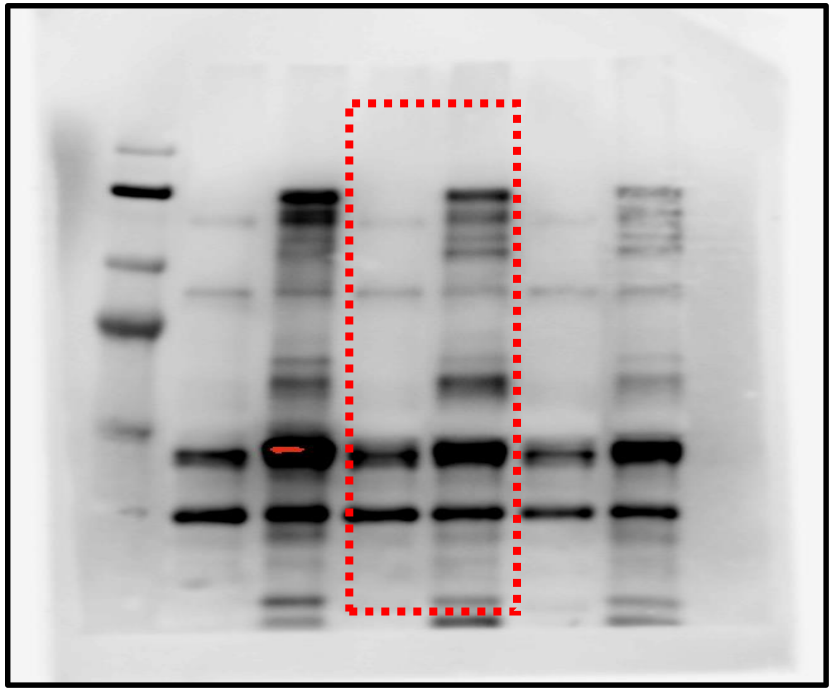

Anti-His

4 x (EV; FLAG-RFP-*Nb*SCLP20b-His-GFP)

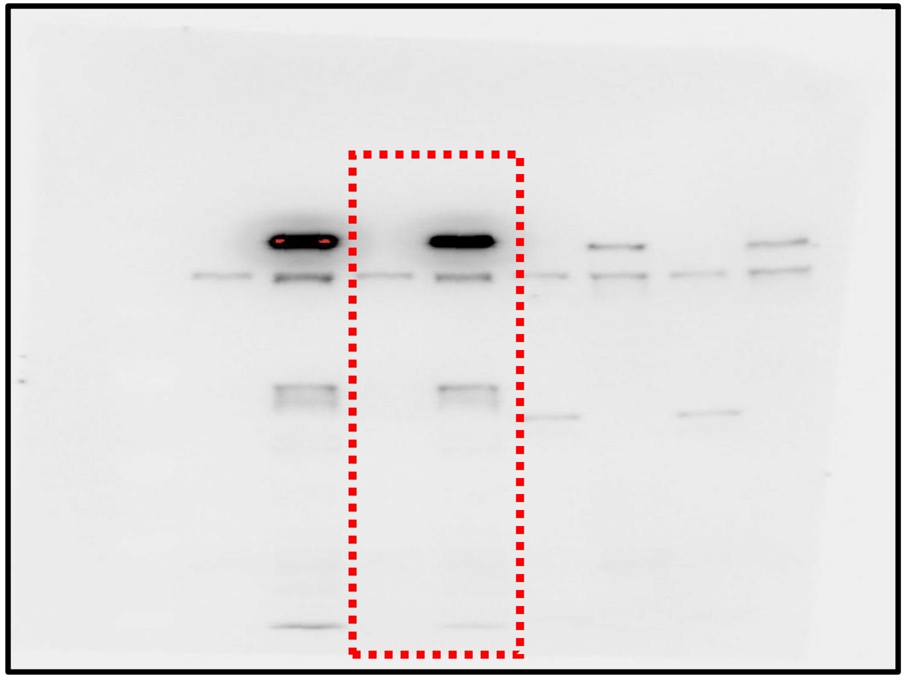

Anti-FLAG

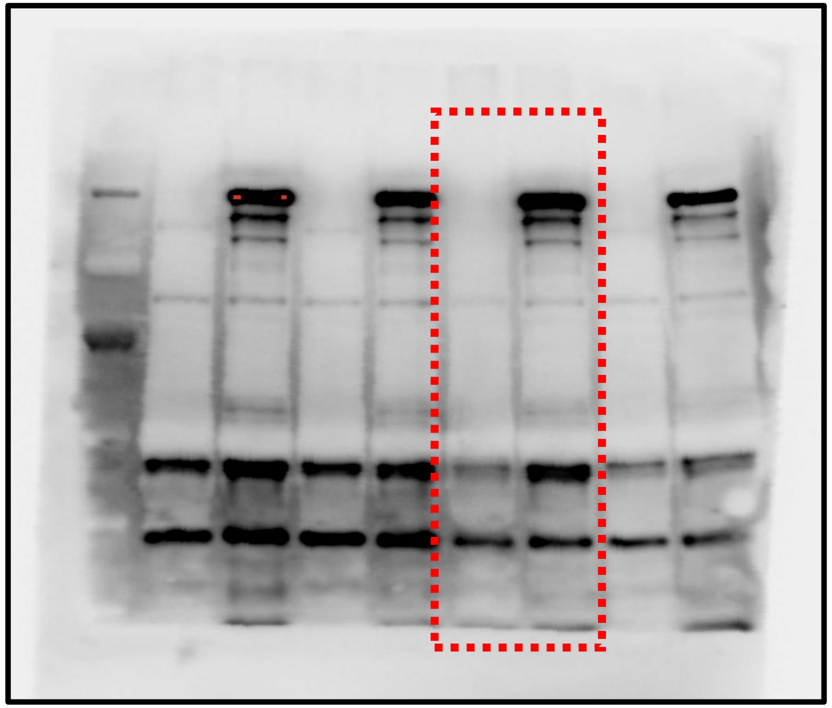

Anti-His

4 x (EV; FLAG-RFP-*Nb*EDA2-His-GFP)

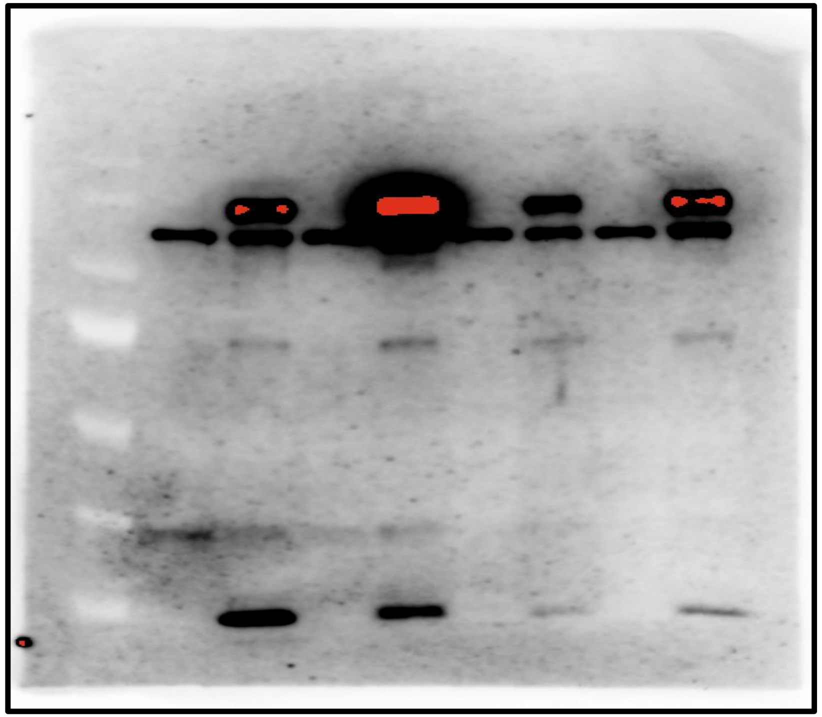

Anti-FLAG

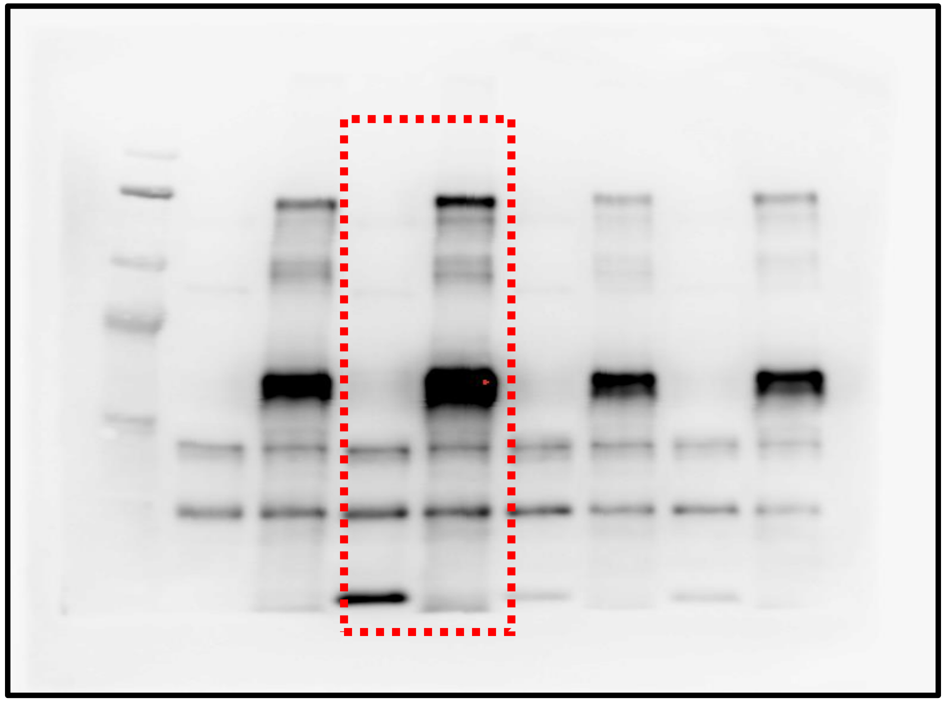

Anti-His

3 x (EV; FLAG-RFP-*Nb*GMCO-His-GFP)

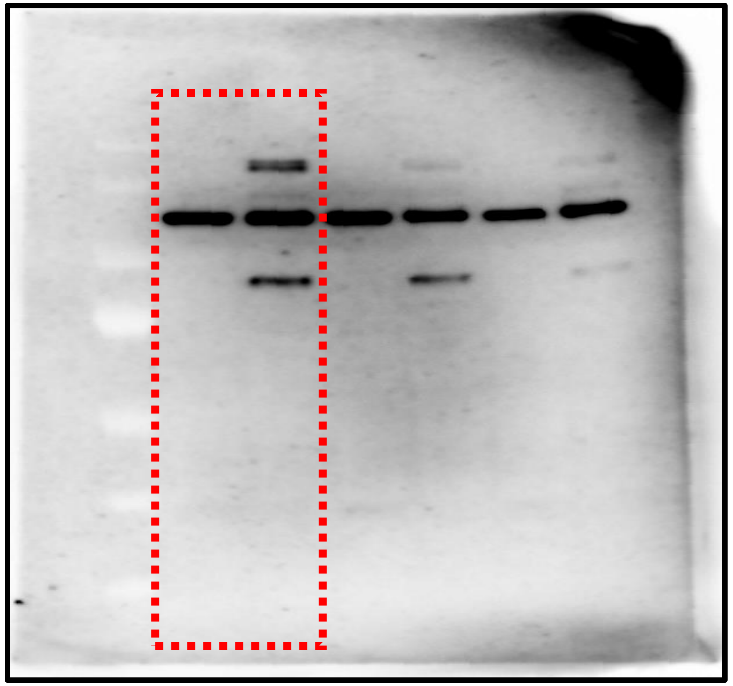

Anti-FLAG

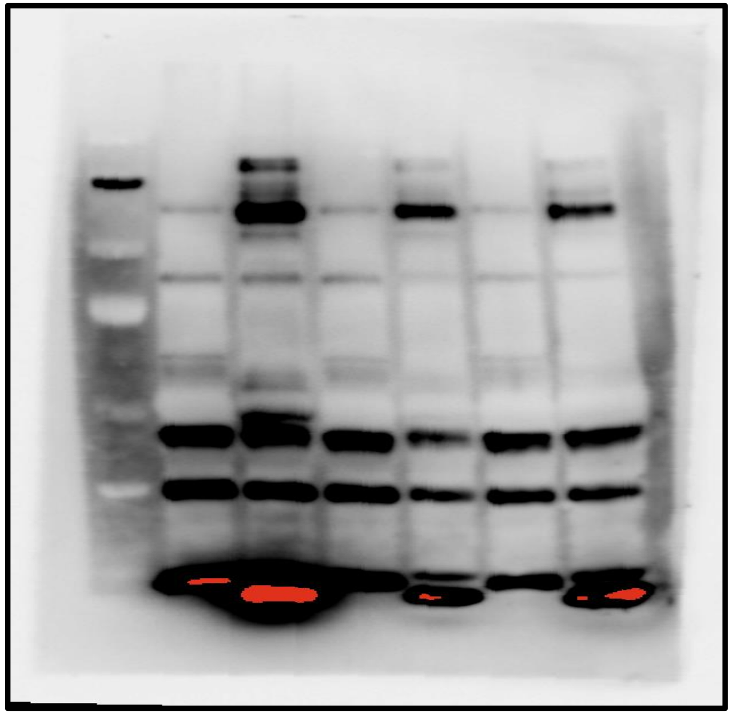

Anti-His

3 x (EV; 2 x FLAG-RFP-*Nb*GMCO-His-GFP)

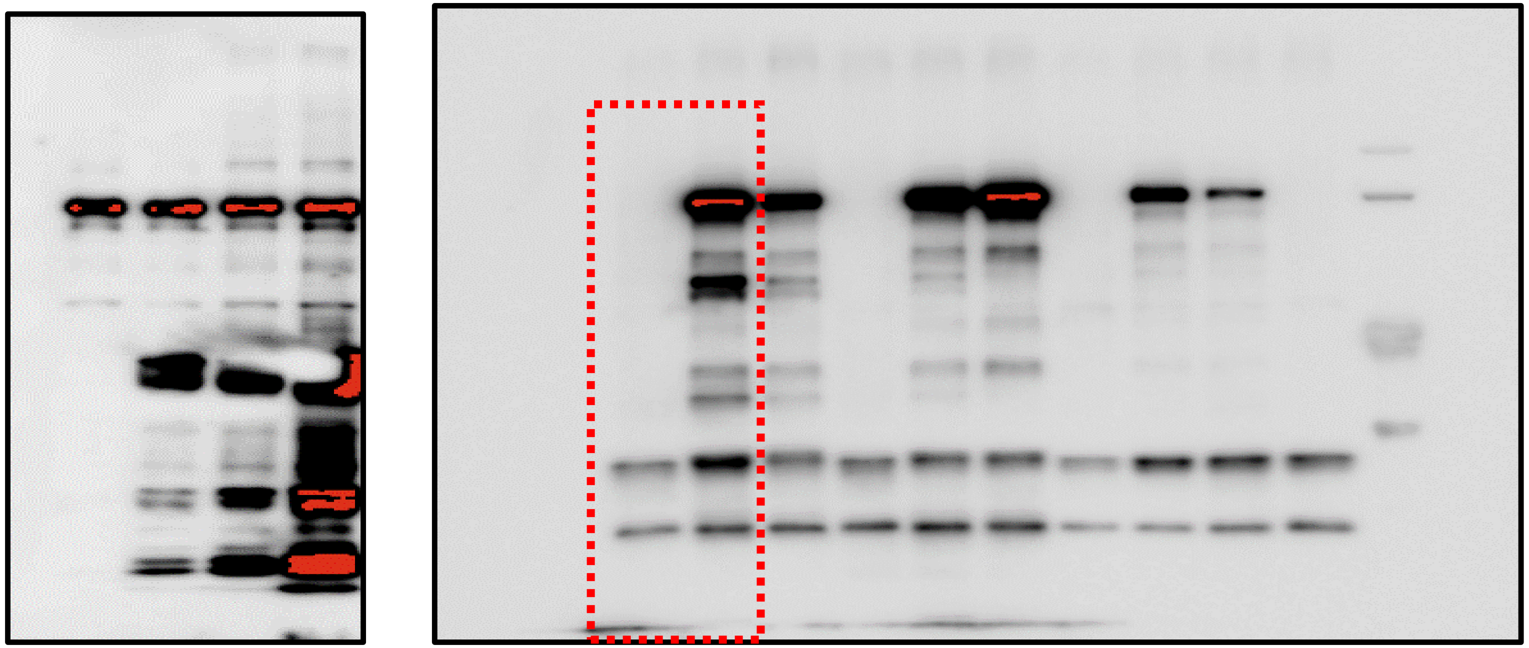

Anti-FLAG *Anti-His*
